# Synthesis of Natural Homoisoflavonoids Having Either 5,7-Dihydroxy-6-methoxy or 7-Hydroxy-5,6-dimethoxy Groups

**DOI:** 10.3390/molecules21081058

**Published:** 2016-08-13

**Authors:** Hyungjun Lee, Yue Yuan, Inmoo Rhee, Timothy W. Corson, Seung-Yong Seo

**Affiliations:** 1 College of Pharmacy and Gachon Institute of Pharmaceutical Sciences, Gachon University, Incheon 21936, Korea; a13079@naver.com (H.L.); wonwol@gmail.com (Y.Y.); 2 Department of Bioscience and Biotechnology, Sejong University, Seoul 05006, Korea; nature@sejong.ac.kr; 3 Departments of Ophthalmology, Eugene and Marilyn Glick Eye Institute, Biochemistry and Molecular Biology, and Pharmacology and Toxicology, Indiana University School of Medicine, Indianapolis, IN 46202, USA; tcorson@iu.edu

**Keywords:** homoisoflavanone, cremastranone, 4-chromanone, 4-chromenone 1,4-reduction

## Abstract

Naturally occurring homoisoflavonoids containing either 5,7-dihydroxy-6-methoxy or 7-hydroxy-5,6-dimethoxy groups such as the antiangiogenic homoisoflavanone, cremastranone, were synthesized via three or four linear steps from the known 4-chromenone. This facile synthesis includes chemoselective 1,4-reduction of 4-chromenone and selective deprotection of 3-benzylidene-4-chromanone a containing C7-benzyloxy group.

## 1. Introduction

**Figure 1:**
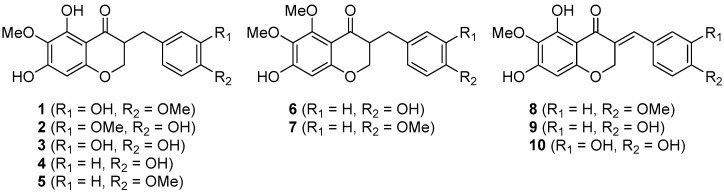
Naturally occurring homoisoflavonoids containing either 5,7-dihydroxy-6-methoxy (i.e., **1**–**5** and **8**–**10**) or 7-hydroxy-5,6-dimethoxy (**6** and **7**).

Recently, the antiangiogenic homoisoflavonoid cremastranone (**1**), isolated from the plants *Muscari armeniacum*, *Chionodoxa luciliae*, *Scilla natalensis*, *Merwilla plumbea*, and *Cremastra appendiculata* was synthesized for the first time by us [1,2,3,4,5,6]. Its naturally occurring congeners (**2**–**10**), which contain either 5,7-dihydroxy-6-methoxy or 7-hydroxy-5,6-dimethoxy groups, have been reported already as shown in Figure 1 and most of them lack common names other than eucomnalin (**9**; autumnalin) and 3,9-dihydroeucomnalin (**4**; 3,9-dihydroautumnalin). Two review articles deal with the natural origins and structures of most homoisoflavonoids [7,8], and thereafter various studies of the homoisoflavonoids have been published [9,10,11]. Nevertheless, there have not been synthetic efforts towards such homoisoflavonoids since the synthesis of **4** and **9** was reported in 1971 [12]. A chemical synthesis of 5,6,7-trisubstituted homoisoflavonoids has the potential to provide a general and expedient entry into a plethora of analogues potentially with interesting biological activities [13,14,15]. Herein we report the first synthesis of cremastranone’s derivatives in three or four steps from the known 4-chromenone, involving a chemoselective 1,4-reduction and manipulation of protecting groups.

## 2. Results and Discussion

**Scheme 1:**
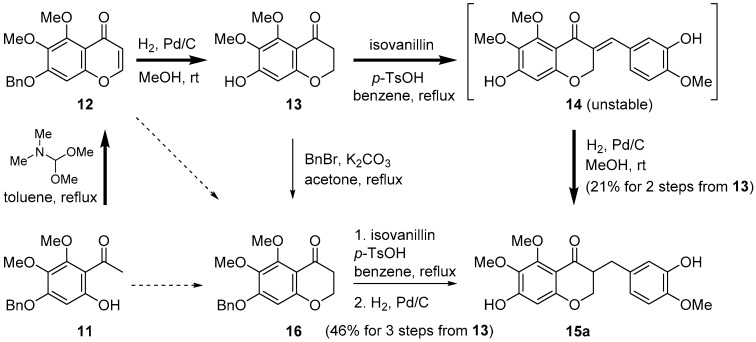
Our previous approach using 5,6,7-trisubstituted 3-benzyl-4-chromanone for the reported synthesis of cremastranone (bold arrow) [14] and the alternative pathway (normal arrow).

In our initial approach to cremastranone (**1**), the acetophenone **11** as starting material was treated with *N*,*N*-dimethylformamide dimethyl acetal, followed by catalytic hydrogenation to afford the 4-chromanone **13** in a good yield (Scheme 1) [14]. However, aldol condensation of **13** with isovanillin gave the unstable benzylidene products (i.e., **14a**) in a low yield; the free OH group on C7 is thought to hamper the general aldol condensation as well as the stability of the desired products. To overcome this drawback, the benzylation of the phenol group on C7 was adopted prior to aldol condensation. The aldol condensation of **16** with isovanillin afforded the desired compound **15a** in good yield.

To improve chemical yield and decrease reaction steps, we focused on the direct conversion of either the acetophenone **11** or the chromenone **12** into the desired chromanone **16**. However, it was reported that the formation of a 4-chromanone from a 2-hydroxyacetophenone (i.e., **11**) is challenging [16]. The direct conversion of **12** to **16** was attempted under some reduction conditions (Table 1) [17]. Even careful catalytic hydrogenation is not likely to control a partial reduction of only the double bond. Among metal hydride reagents, the reduction using 1.5 equivalent of NaBH_4_ did not proceed over 5 h, forming only a small amount of 4-chromanol **17** (<10%). Treatment with excess NaBH_4_ afforded only 4-chromanol **17** in 50% yield (80% based on the recovered **12**). Diisobutylaluminum hydride (DIBAL) reduction at −60 °C led to the nonselective generation of **16** (20%) and **17** (20%) in low yields. To our delight, the reduction by LiAlH_4_ underwent only 1,4-addition of hydride without over-reduction quantitatively and had the advantage of being reproducible on a gram scale.

**Scheme 2:**
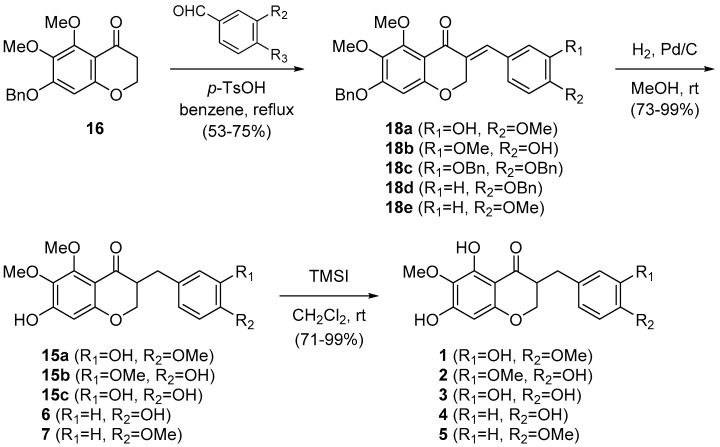
Synthesis of natural products, 5,6,7-trisubstituted 3-benzyl-4-chromanones.

With **16** in hand, we turned to the synthesis of natural 3-benzyl-4-chromanones containing 5,7-dihydroxy-6-methoxy and 7-hydroxy-5,6-dimethoxy groups, similar to cremastranone (Scheme 2). To accomplish this, the 4-chromanone **16** was coupled with a 3,4-disubstituted benzaldehyde (i.e., vanillin or 3,4-dibenzyloxybenzaldehyde) or a 4-substituted benzaldehyde (i.e., 4-methoxybenzaldehyde or 4-benzyloxybenzaldehyde) under acidic conditions to afford 3-benzylidene-4-chromanones (**18a**–**18e**). Catalytic hydrogenation of **18b** and **18c**, followed by trimethylsilyl iodide (TMSI)-promoted C5-demethylation of **15b** and **15c** provided the desired **2** and **3**, respectively. In a similar manner, using catalytic hydrogenation **18d** and **18e** were transformed to **6** and **7**, which were treated with TMSI to provide **4** and **5**, respectively. The spectroscopic properties (^1^H- and ^13^C-NMR, mass spectrometry) of synthetic **2**, **3**, **4** [2], **5** [5], **6** [18], and **7** [19] were compatible with those of all the natural products (see Supplementary Materials).

On the other hand, for the synthesis of three 3-benzylidene-4-chromanones (**8**–**10**) containing a 5,7-dihydroxy-6-methoxy group, the C7-benzyl group of the synthesized benzylidene intermediates (**18c**–**18e**) should be removed while keeping the C3–C9 double bond intact. With **18e** in hand, we next attempted the debenzylation on C7 as shown in Table 2. Whereas the general condition of catalytic hydrogenation under H_2_ and Pd/C afforded the saturated 3-benzyl-4-chromanone such as the synthesis of **7**, a small amount (3 mol %) of Pd/C catalyst and a short reaction time (5 min) led to the desired product **19** in moderate yield. TMSI and HBr selectively provided **19**, which was demethylated only on the C5 position. But no product formation was observed with the treatment of TiCl and lithium naphthalenide. Unfortunately, the two and three benzyl groups of **18c** and **18d**, respectively, could not be cleaved simultaneously before the saturation of the C3–C9 double bond, using various conditions of catalytic hydrogenation.

**Scheme 3:**
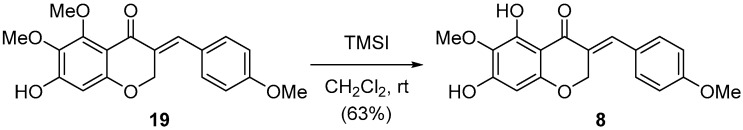
Synthesis of natural 3-benzylidene-4-chromanone **8**.

Finally, the debenzylated compound **19** was treated with TMSI to provide the desired **8** in good yield as shown in Scheme 3. The spectroscopic properties (^1^H- and ^13^C-NMR, mass spectrometry) of synthetic **8** were compatible with those of the natural product **8** [20]. Having failed to access **9** and **10** via the debenzylation strategy, further studies on other protecting groups on C7 such as PMB (4-methoxybenzyl), MOM (methoxymethyl) and Ms (methanesulfonyl) instead of benzyl ether are under investigation.

## 3. Materials and Methods

### 3.1. General Information

All starting materials and reagents were obtained from commercial suppliers and were used without further purification. Air- and moisture-sensitive reactions were performed under an argon atmosphere. Flash column chromatography was performed using silica gel 60 (230–400 mesh, Merck KGaA, Darmstadt, Germany) with the indicated solvents. Thin-layer chromatography was performed using 0.25 mm silica gel plates (Merck KGaA). ^1^H- and ^13^C-NMR spectra were recorded on a Bruker 600 MHz spectrometer as solutions in deuteriochloroform (CDCl_3_) or methanol-*d*_4_ (Cambridge Isotope Laboratory, Andover, MA, USA). ^1^H-NMR data were reported in the order of chemical shift, multiplicity (s, singlet; d, doublet; t, triplet; m, multiplet; and/or multiple resonances), number of protons, and coupling constant (*J*) in hertz (Hz). High-resolution mass spectra (HRMS) were recorded on a JEOL JMS-700 (EI) (JEOL Ltd., Tokyo, Japan) and an Agilent 6530 Q-TOF LC/MS/MS system (ESI) (Agilent Technologies, Inc., Santa Clara, CA, USA).

### 3.2. Synthesis

*7-(Benzyloxy)-5,6-dimethoxychroman-4-one* (**16**). To a solution of the acetophenone (**11**) (100 mg, 0.33 mmol) in toluene (2.0 mL) was added *N*,*N*-dimethylformamide dimethyl acetal (52 μL, 0.39 mmol). After stirring for 18 h at 80 °C, the mixture was cooled to 0 °C and c-HCl (0.2 mL) was added. After stirring for 1 h at 50 °C. The reaction mixture was diluted with ethyl acetate and the organic phase was washed with water and brine, and dried over anhydrous MgSO_4_. The solvent was removed under reduced pressure and purified by flash column chromatography on silica gel (ethyl acetate:*n*-hexane = 1:2) to afford 7-(benzyloxy)-5,6-dimethoxy-4*H*-chromen-4-one (**12**) (101 mg, 97%). ^1^H-NMR (CDCl_3_, 600 MHz) δ 7.63 (d, 1H, *J* = 6.0 Hz), 7.46–7.40 (m, 4H), 7.37 (t, 1H, *J* = 6.6 Hz), 6.71 (s, 1H), 6.17 (d, 1H, *J* = 6.0 Hz), 5.20 (s, 2H), 3.97 (s, 3H), 3.92 (s, 3H); ^13^C-NMR (150 MHz, CDCl_3_) δ 176.2, 156.8, 154.6, 152.9, 140.7, 135.5, 128.8, 128.4, 127.2, 114.2, 113.8, 97.6, 70.9, 62.1, 61.5, 30.9. To a stirred solution of 4-chromenone (**12**) (10 mg, 0.03 mmol) in dry THF and Et_2_O (1:1) at −60 °C a solution of LiAlH_4_ in dry THF was added under N_2_ atmosphere. After stirring for 5 min, the reaction mixture was diluted with ethyl acetate and the organic phase was washed with water and brine, and dried over anhydrous MgSO_4_. The solvent was removed under reduced pressure and purified by flash column chromatography on silica gel (ethyl acetate:*n*-hexane = 1:2) to afford the 7-(benzyloxy)-5,6-dimethoxychroman-4-one (**16**) (10 mg, 99%). ^1^H-NMR (CDCl_3_, 600 MHz) δ 7.43–7.37 (m, 4H), 7.35–7.33 (m, 1H), 6.29 (s, 1H), 5.13 (s, 1H), 4.42 (t, 2H, *J* = 6.0 Hz), 3.92 (s, 3H), 3.82 (s, 3H), 2.71 (t, 2H, *J* = 6.0 Hz); ^13^C-NMR (150 MHz, CDCl_3_) δ 189.1, 159.8, 158.4, 154.4, 137.7, 135.8, 128.7, 128.2, 127.2, 109.8, 97.4, 70.6, 66.8, 61.6, 61.3, 38.7.

*(E)-7-(Benzyloxy)-3-(3-hydroxy-4-methoxybenzylidene)-5,6-dimethoxychroman-4-one* (**18a**). To a solution of the 4-chromanone (**16**) (80 mg, 0.26 mmol) in benzene (5 mL) was added 3-hydroxy-4-methoxybenzaldehyde (58 mg, 0.38 mmol) and *p*-toluenesulfonic acid (7 mg, 0.03 mmol) at 0 °C. The reaction mixture was refluxed for 12 h. After cooling to room temperature, the reaction mixture was concentrated under reduced pressure. The residue was purified by flash column chromatography on silica gel (ethyl acetate:*n*-hexane = 1:2) to afford the resulting 3-benzylidene-4-chromanone (**18a**) (60 mg, 53%). ^1^H-NMR (600 MHz, CDCl_3_) δ 7.73 (s, 1H), 7.42–7.38 (m, 4H), 7.35–7.33 (m, 1H), 6.90 (d, 1H, *J* = 8.4 Hz), 6.86–6.83 (m, 2H), 6.30 (s, 1H), 5.22 (d, 2H, *J* = 1.8 Hz), 5.13 (s, 2H), 3.99 (s, 3H), 3.94 (s, 3H), 3.85 (s, 3H); ^13^C-NMR (150 MHz, CDCl_3_) δ 179.6, 159.2, 158.2, 154.9, 147.6, 145.5, 138.1, 136.3, 135.8, 130.1, 128.7, 128.2, 128.0, 127.2, 123.2, 115.8, 110.8, 110.6, 97.5, 70.6, 67.6, 61.7, 61.3, 56.0; HRMS (EI): mass calculated for C_26_H_24_O_7_ [M^+^], 448.1522; found, 448.1521.

*(E)-7-(Benzyloxy)-3-(4-hydroxy-3-methoxybenzylidene)-5,6-dimethoxychroman-4-one* (**18b**). To a solution of the 4-chromanone (**16**) (80 mg, 0.26 mmol) in benzene (5 mL) was added 4-hydroxy-3-methoxybenzaldehyde (58 mg, 0.38 mmol) and *p*-toluenesulfonic acid (7 mg, 0.03 mmol) at 0 °C. The reaction mixture was refluxed for 12 h. After cooling to room temperature, the reaction mixture was concentrated under reduced pressure. The residue was purified by flash column chromatography on silica gel (ethyl acetate:*n*-hexane = 1:2) to afford the resulting 3-benzylidene-4-chromanone (**18b**) (65 mg, 57%). ^1^H-NMR (600 MHz, CDCl_3_) δ 7.76 (s, 1H), 7.43–7.38 (m, 4H), 7.35–7.32 (m, 1H), 6.97 (d, 1H, *J* = 8.4 Hz), 6.82 (d, 1H, *J* = 1.8 Hz), 6.79 (dd, 1H, *J* = 7.8 and 1.8 Hz), 6.30 (s, 1H), 5.22 (d, 2H, *J* = 1.2 Hz), 5.13 (s, 2H), 3.99 (s, 3H), 3.91 (s, 3H), 3.85 (s, 3H); ^13^C-NMR (150 MHz, CDCl_3_) δ 179.5, 159.1, 158.2, 154.9, 146.9, 146.5, 138.1, 136.5, 135.8, 129.8, 128.7, 128.2, 127.2, 127.1, 123.7, 114.6, 112.7, 110.8, 97.5, 70.6, 67.6, 61.7, 61.3, 56.0; HRMS (EI): mass calculated for C_26_H_24_O_7_ [M^+^], 448.1522; found, 448.1521.

*(E)-7-(Benzyloxy)-3-(3,4-bis(benzyloxy)benzylidene)-5,6-dimethoxychroman-4-one* (**18c**). To a solution of the 4-chromanone (**16**) (80 mg, 0.25 mmol) in benzene (5 mL) was added 3,4-bis(benzyloxy)benzaldehyde (120 mg, 0.38 mmol) and *p*-toluenesulfonic acid (7 mg, 0.03 mmol) at 0 °C. The reaction mixture was refluxed for 12 h. After cooling to room temperature, the reaction mixture was concentrated under reduced pressure. The residue was purified by flash column chromatography on silica gel (ethyl acetate:*n*-hexane = 1:2) to afford the resulting 3-benzylidene-4-chromanone (**18c**) (55 mg, 36%). ^1^H-NMR (600 MHz, CDCl_3_) δ 7.68 (s, 1H), 7.46–7.31 (m, 15H), 6.96 (d, 2H, *J* = 9.0 Hz), 6.82 (dd, 2H, *J* = 4.8 and 3.0 Hz), 6.29 (s, 1H), 5.22 (s, 2H), 5.19 (s, 2H), 5.13 (s, 2H), 5.04 (d, 2H, *J* = 1.8 Hz), 3.98 (s, 3H), 3.85 (s, 3H); ^13^C-NMR (150 MHz, CDCl_3_) δ 179.5, 159.1, 158.2, 154.9, 150.0, 148.4, 138.1, 136.8, 136.7, 136.1, 135.8, 130.1, 128.7, 128.6, 128.6, 128.4, 128.2, 128.0, 127.9, 127.5, 127.2, 127.2, 124.1, 116.9, 114.2, 110.8, 97.5, 71.4, 71.0, 70.6, 67.5, 61.7, 61.3; HRMS (EI): mass calculated for C_39_H_34_O_7_ [M^+^], 614.2305; found, 614.2308.

*(E)-7-(Benzyloxy)-3-(4-(benzyloxy)benzylidene)-5,6-dimethoxychroman-4-one* (**18d**). To a solution of the 4-chromanone (**16**) (121 mg, 0.39 mmol) in benzene (5 mL) was added 4-(benzyloxy)benzaldehyde (122 mg, 0.58 mmol) and *p*-toluenesulfonic acid (7 mg, 0.03 mmol) at 0 °C. The reaction mixture was refluxed for 12 h. After cooling to room temperature, the reaction mixture was concentrated under reduced pressure. The residue was purified by flash column chromatography on silica gel (ethyl acetate:*n*-hexane = 1:2) to afford the resulting 3-benzylidene-4-chromanone (**18d**) (104 mg, 54%). ^1^H-NMR (600 MHz, CDCl_3_) δ 7.78 (s, 1H), 7.44–7.38 (m, 8H), 7.35–7.33 (m, 2H), 7.24 (d, 2H, *J* = 9.0 Hz), 7.24 (dd, 2H, *J* = 6.6 and 1.8 Hz), 6.30 (s, 1H), 5.21 (d, 2H, *J* = 1.8 Hz), 5.13 (s, 2H), 5.11 (s, 2H), 4.00 (s, 3H), 3.85 (s, 3H); ^13^C-NMR (150 MHz, CDCl_3_) δ 179.6, 159.6, 159.1, 158.2, 154.9, 138.1, 136.4, 136.1, 135.8, 131.7, 129.8, 128.7, 128.7, 128.2, 128.1, 127.5, 127.4, 127.2, 115.0, 110.8, 97.5, 70.6, 70.1, 67.6, 61.7, 61.3; HRMS (EI): mass calculated for C_32_H_28_O_6_ [M^+^], 508.1886; found, 508.1885.

*(E)-7-(Benzyloxy)-5,6-dimethoxy-3-(4-methoxybenzylidene)chroman-4-one* (**18e**). To a solution of the 4-chromanone (**16**) (82 mg, 0.26 mmol) in benzene (5 mL) was added 4-methoxybenzaldehyde (50 μL, 0.39 mmol) and *p*-toluenesulfonic acid (7 mg, 0.03 mmol) at 0 °C. The reaction mixture was refluxed for 12 h. After cooling to room temperature, the reaction mixture was concentrated under reduced pressure. The residue was purified by flash column chromatography on silica gel (ethyl acetate:*n*-hexane = 1:2) to afford the resulting 3-benzylidene-4-chromanone (**18e**) (78 mg, 75%). ^1^H-NMR (600 MHz, CDCl_3_) δ 7.78 (s, 1H), 7.44–7.38 (m, 8H), 7.35–7.33 (m, 2H), 7.24 (d, 2H, *J* = 9.0 Hz), 7.24 (dd, 2H, *J* = 6.6 and 1.8 Hz), 6.30 (s, 1H), 5.21 (d, 2H, *J* = 1.8 Hz), 5.13 (s, 2H), 5.11 (s, 2H), 4.00 (s, 3H), 3.85 (s, 3H); ^13^C-NMR (150 MHz, CDCl_3_) δ 179.6, 159.6, 159.1, 158.2, 154.9, 138.1, 136.4, 136.1, 135.8, 131.7, 129.8, 128.7, 128.7, 128.2, 128.1, 127.5, 127.4, 127.2, 115.0, 110.8, 97.5, 70.6, 70.1, 67.6, 61.7, 61.3; HRMS (EI): mass calculated for C_26_H_24_O_6_ [M^+^], 432.1573; found, 432.1573.

*7-Hydroxy-3-(3-hydroxy-4-methoxybenzyl)-5,6-dimethoxychroman-4-one* (**15a**). A solution of the 3-benzylidene-4-chromanone (**18a**) (35 mg, 0.07 mmol) and 10% Pd/C (10 mg) in MeOH was placed under an atmosphere of hydrogen. After stirring for 1 h, the reaction mixture was diluted with ethyl acetate, filtered through a Celite pad, and concentrated under reduced pressure. The residue was purified by flash column chromatography on silica gel (ethyl acetate:*n*-hexane = 1:1) to afford the 3-benzyl-4-chromanone (**15a**) (22 mg, 87%). ^1^H-NMR (600 MHz, CD_3_OD) δ 6.82 (d, 1H, *J* = 14.4 Hz), 6.67 (d, 1H, *J* = 1.8 Hz), 6.63 (dd, 1H, *J* = 8.4 and 2.4 Hz), 6.16 (s, 1H), 4.21 (dd, 1H, *J* = 11.4 and 4.2 Hz), 4.04 (dd, 1H, *J* = 11.4 and 7.2 Hz), 3.82 (s, 3H), 3.79 (s, 3H), 3.75 (s, 3H), 3.00 (dd, 1H, *J* = 13.2 and 4.2 Hz), 2.66–2.60 (m, 1H), 2.58 (dd, 1H, *J* = 13.8 and 10.8 Hz); ^13^C-NMR (150 MHz, CD_3_OD) δ 192.4, 160.0, 158.5, 154.4, 146.3, 146.2, 136.4, 131.2, 119.9, 115.6, 111.5, 107.3, 99.1, 68.6, 60.4, 60.1, 55.0, 48.2, 32.0; HRMS (ESI): mass calcd for C_19_H_20_O_7_ [M + H^+^], 361.1281; found 361.1270.

*7-Hydroxy-3-(4-hydroxy-3-methoxybenzyl)-5,6-dimethoxychroman-4-one* (**15b**). A solution of the 3-benzylidene-4-chromanone (**18b**) (21 mg, 0.05 mmol) and 10% Pd/C (5 mg) in MeOH was placed under an atmosphere of hydrogen. After stirring for 1 h, the reaction mixture was diluted with ethyl acetate, filtered through a Celite pad and concentrated under reduced pressure. The residue was purified by flash column chromatography on silica gel (ethyl acetate:*n*-hexane = 1:1) to afford the 3-benzyl-4-chromanone (**15b**) (15 mg, 73%). ^1^H-NMR (600 MHz, CDCl_3_) δ 6.84 (d, 1H, *J* = 8.4 Hz), 6.73–6.69 (m, 2H), 6.31 (s, 1H), 4.26 (dd, 1H, *J* = 11.4 and 4.2 Hz), 4.10 (dd, 1H, *J* = 11.4 and 7.2 Hz), 3.91 (s, 3H), 3.91 (s, 3H), 3.87 (s, 3H), 3.16 (dd, 1H, *J* = 13.8 and 4.2 Hz), 2.73 (m, 1H), 2.65–2.61 (m, 1H); ^13^C-NMR (150 MHz, CDCl_3_) δ 191.6, 159.8, 155.5, 153.5, 146.5, 144.2, 135.2, 130.2, 121.9, 114.3, 111.4, 108.7, 98.8, 68.8, 61.5, 61.4, 55.9, 48.6, 32.6; HRMS (EI): mass calculated for C_19_H_20_O_7_ [M^+^], 360.1209; found, 360.1208.

*3-(3,4-Dihydroxybenzyl)-7-hydroxy-5,6-dimethoxychroman-4-one* (**15c**). A solution of the 3-benzylidene-4-chromanone (**18c**) (20 mg, 33 μmol) and 10% Pd/C (3.5 mg) in MeOH was placed under an atmosphere of hydrogen. After stirring for 1 h, the reaction mixture was diluted with ethyl acetate, filtered through a Celite pad and concentrated under reduced pressure. The residue was purified by flash column chromatography on silica gel (ethyl acetate:*n*-hexane = 1:1) to afford the 3-benzyl-4-chromanone (**15c**) (11 mg, 99%). ^1^H-NMR (600 MHz, CDCl_3_) δ 6.79 (d, 1H, *J* = 8.4 Hz), 6.77 (d, 1H, *J* = 2.4 Hz), 6.63 (dd, 1H, *J* = 8.4 and 1.8 Hz), 6.30 (s, 1H), 4.26 (dd, 1H, *J* = 11.4 and 4.2 Hz), 4.09 (dd, 1H, *J* = 8.4 and 4.2 Hz), 3.90 (s, 3H), 3.89 (s, 3H), 3.08 (dd, 1H, *J* = 13.8 and 4.2 Hz), 2.70–2.67 (m, 1H), 2.62 (dd, 1H, *J* = 13.8 and 10.8 Hz); ^13^C-NMR (150 MHz, CDCl_3_) δ 192.2, 159.9, 155.9, 153.5, 143.8, 142.5, 135.2, 131.0, 121.6, 116.0, 115.3, 108.5, 98.9, 68.8, 61.5, 61.4, 48.5, 32.4; HRMS (EI): mass calculated for C_18_H_18_O_7_ [M^+^], 346.1053; found, 346.1056.

*7-Hydroxy-3-(4-hydroxybenzyl)-5,6-dimethoxychroman-4-one* (**6**). A solution of the 3-benzylidene-4-chromanone (**18d**) (23 mg, 0.05 mmol) and 10% Pd/C (5 mg) in MeOH was placed under an atmosphere of hydrogen. After stirring for 1 h, the reaction mixture was diluted with ethyl acetate, filtered through a Celite pad and concentrated under reduced pressure. The residue was purified by flash column chromatography on silica gel (ethyl acetate:*n*-hexane = 1:1) to afford the 3-benzyl-4-chromanone (**6**) (14 mg, 94%). ^1^H-NMR (600 MHz, CDCl_3_) δ 7.09 (d, 2H, *J* = 8.4 Hz), 6.78 (d, 2H, *J* = 8.4 Hz), 6.30 (s, 1H), 4.62 (dd, 1H, *J* = 11.4 and 4.2 Hz), 4.08 (dd, 1H, *J* = 11.4 and 7.2 Hz), 3.91 (s, 3H), 3.90 (s, 3H), 3.15 (dd, 1H, *J* = 13.8 and 4.2 Hz), 2.73–2.69 (m, 1H), 2.66 (dd, 1H, *J* = 13.8 and 10.2 Hz); ^13^C-NMR (150 MHz, CDCl_3_) δ 191.7, 159.8, 155.6, 154.3, 153.5, 135.2, 130.3, 130.3, 115.4, 108.6, 98.8, 68.8, 61.5, 61.4, 48.5, 32.0; HRMS (EI): mass calculated for C_18_H_18_O_6_ [M^+^], 330.1103; found, 330.1102.

*7-Hydroxy-5,6-dimethoxy-3-(4-methoxybenzyl)chroman-4-one* (**7**). A solution of the 3-benzylidene-4-chromanone (**18e**) (23 mg, 0.05 mmol) and 10% Pd/C (5 mg) in MeOH was placed under an atmosphere of hydrogen. After stirring for 1 h, the reaction mixture was diluted with ethyl acetate, filtered through a Celite pad and concentrated under reduced pressure. The residue was purified by flash column chromatography on silica gel (ethyl acetate:*n*-hexane = 1:2) to afford the 3-benzyl-4-chromanone (**7**) (13 mg, 80%). ^1^H-NMR (600 MHz, CD_3_COCD_3_) δ 7.07–7.05 (m, 2H), 6.76–6.73 (m, 2H), 6.09 (dd, 1H, *J* = 4.8 and 3.0 Hz), 4.14 (d, 1H, *J* = 4.2 Hz), 3.94 (d, 1H, *J* = 8.4 Hz), 3.71 (s, 3H), 3.71–3.62 (m, 6H), 3.00–3.98 (m, 1H), 2.62–2.61 (m, 1H), 2.53–2.49 (m, 1H); ^13^C-NMR (150 MHz, CDCl_3_) δ 190.0, 159.6, 158.5, 157.1, 154.7, 136.3, 160.8, 130.8, 130.1, 113.9, 108.5, 99.1, 69.1, 60.8, 60.6, 54.6, 48.3, 31.5; HRMS (EI): mass calculated for C_19_H_20_O_6_ [M^+^], 344.1260; found, 344.1257.

*7-Hydroxy-3-(3-hydroxy-4-methoxybenzyl)-5,6-dimethoxychroman-4-one* (**2**). To a solution of the 7-hydroxy-5,6-dimethoxy-4-chromanone (**15b**) (10 mg, 0.03 mmol) in CH_2_Cl_2_ (5 mL) was added TMSI (5 μL, 0.09 mmol) at 0 °C for 1 h. The mixture was concentrated in vacuo. The residue was purified by flash column chromatography on silica gel (ethyl acetate:*n*-hexane = 1:2) to afford the 5,7-dihydroxy-6-methoxy-4-chromanone (**2**) (7.1 mg, 71%). ^1^H-NMR (600 MHz, CD_3_OD) δ 6.81 (d, 1H, *J* = 1.8 Hz), 6.73 (d, 1H, *J* = 7.8 Hz), 6.67 (dd, 1H, *J* = 8.4 and 1.8 Hz), 5.90 (s, 1H), 4.25 (dd, 1H, *J* = 11.4 and 4.2 Hz), 4.09 (dd, 1H, *J* = 11.4 and 7.2 Hz), 3.83 (s, 3H), 3.77 (s, 3H), 3.12 (dd, 1H, *J* = 13.8 and 4.8 Hz), 2.86–2.81 (m, 1H), 2.68 (d, 1H, *J* = 10.2 Hz); ^13^C-NMR (150 MHz, CD_3_OD) δ 199.3, 160.1, 159.3, 156.0, 148.2, 145.5, 130.0, 129.6, 121.9, 115.4, 112.8, 102.1, 95.0, 69.5, 60.1, 55.5, 47.3, 32.7; HRMS (EI): mass calculated for C_18_H_18_O_7_ [M^+^], 346.1053; found, 346.1054.

*3-(3,4-Dihydroxybenzyl)-5,7-dihydroxy-6-methoxychroman-4-one* (**3**). To a solution of the 7-hydroxy-5,6-dimethoxy-4-chromanone (**15c**) (20 mg, 0.03 mmol) in CH_2_Cl_2_ (5 mL) was added TMSI (5 μL, 0.09 mmol) at 0 °C for 1 h. The mixture was concentrated in vacuo. The residue was purified by flash column chromatography on silica gel (ethyl acetate:*n*-hexane = 1:2) to afford the 5,7-dihydroxy-6-methoxy-4-chromanone (**3**) (12 mg, 99%). ^1^H-NMR (600 MHz, CD_3_OD) δ 6.71 (d, 1H, *J* = 8.4 Hz), 6.67 (d, 1H, *J* = 2.4 Hz), 6.55 (dd, 1H, *J* = 7.8 and 1.8 Hz), 5.91 (s, 1H), 4.24 (dd, 1H, *J* = 11.4 and 4.2 Hz), 4.08 (dd, 1H, *J* = 11.4 and 7.2 Hz), 3.77 (s, 3H), 3.05 (dd, 1H, *J* = 13.8 and 4.2 Hz), 2.80–2.75 (m, 1H), 2.60 (dd, 1H, *J* = 13.8 and 10.2 Hz); ^13^C-NMR (150 MHz, CD_3_OD) δ 200.0, 160.5, 159.9, 156.7, 146.2, 144.9, 130.6, 130.2, 121.2, 116.9, 116.2, 102.8, 95.5, 70.0, 60.7, 47.9, 33.0; HRMS (EI): mass calculated for C_17_H_16_O_7_ [M^+^], 332.0896; found, 332.0898.

*5,7-Dihydroxy-3-(4-hydroxybenzyl)-6-methoxychroman-4-one* (**4**). To a solution of the 7-hydroxy-5,6-dimethoxy-4-chromanone (**6**) (10 mg, 0.03 mmol) in CH_2_Cl_2_ (5 mL) was added TMSI (5 μL, 0.09 mmol) at 0 °C for 1 h. The mixture was concentrated in vacuo. The residue was purified by flash column chromatography on silica gel (ethyl acetate:*n*-hexane = 1:2) to afford the resulting 5,7-dihydroxy-6-methoxy-4-chromanone (**4**) (9.8 mg, 86%). ^1^H-NMR (600 MHz, CD_3_OD) δ 7.06 (d, 2H, *J* = 8.4 Hz), 6.73 (d, 2H, *J* = 8.4 Hz), 5.90 (s, 1H), 4.23 (dd, 1H, *J* = 11.4 and 4.2 Hz), 4.07 (dd, 1H, *J* = 10.8 and 7.2 Hz), 3.77 (s, 3H), 3.10 (dd, 1H, *J* = 13.8 and 4.8 Hz), 2.82–2.77 (m, 1H), 2.67 (dd, 1H, *J* = 13.8 and 10.2 Hz); ^13^C-NMR (150 MHz, CD_3_OD) δ 199.8, 160.4, 159.7, 156.8, 156.5, 130.7, 130.0, 129.7, 116.0, 102.5, 95.4, 69.8, 60.5, 47.8, 32.6; HRMS (EI): mass calculated for C_17_H_16_O_6_ [M^+^], 316.0947; found, 316.0945.

*5,7-Dihydroxy-6-methoxy-3-(4-methoxybenzyl)chroman-4-one* (**5**). To a solution of the 7-hydroxy-5,6-dimethoxy-4-chromanone (**7**) (19 mg, 0.06 mmol) in CH_2_Cl_2_ (5 mL) was added TMSI (10 μL, 0.18 mmol) at 0 °C for 1 h. The mixture was concentrated in vacuo. The residue was purified by flash column chromatography on silica gel (ethyl acetate:*n*-hexane = 1:2) to afford the resulting 5,7-dihydroxy-6-methoxy-4-chromanone (**5**) (15 mg, 80%). ^1^H-NMR (600 MHz, CD_3_OD) δ 7.15 (d, 2H, *J* = 8.4 Hz), 6.86 (d, 2H, *J* = 8.4 Hz), 5.90 (s, 1H), 4.23 (dd, 1H, *J* = 11.4 and 4.2 Hz), 4.06 (dd, 1H, *J* = 11.4 and 7.8 Hz), 3.77 (s, 3H), 3.76 (s, 3H), 3.13 (dd, 1H, *J* = 13.8 and 4.8 Hz), 2.84–2.79 (m, 1H), 2.70 (dd, 1H, *J* = 13.8 and 10.2 Hz); ^13^C-NMR (150 MHz, CD_3_OD) δ 200.1, 160.7, 160.1, 160.0, 156.9, 131.4, 131.2, 130.4, 115.1, 103.0, 95.8, 70.3, 61.0, 55.7, 48.1, 32.9; HRMS (EI): mass calculated for C_18_H_18_O_6_ [M^+^], 330.1103; found, 330.1102.

*(E)-7-Hydroxy-5,6-dimethoxy-3-(4-methoxybenzylidene)chroman-4-one* (**19**). A solution of the 3-(4-methoxybenzylidene)-4-chromanone (**18e**) (37 mg, 0.09 mmol) and 3% Pd/C (4.8 mg) in MeOH was placed under an atmosphere of hydrogen. After stirring for 5 min, the reaction mixture was diluted with ethyl acetate, filtered through a Celite pad and concentrated under reduced pressure. The residue was purified by flash column chromatography on silica gel (ethyl acetate:*n*-hexane = 1:3) to afford the debenzylated 3-(4-methoxybenzylidene)-4-chromanone (**19**) (11 mg, 35%). ^1^H-NMR (600 MHz, CDCl_3_) δ 7.78 (s, 1H), 7.26 (d, 2H, *J* = 7.8 Hz), 6.96 (d, 2H, *J* = 9.0 Hz), 6.31 (s, 1H), 5.22 (d, 2H, *J* = 1.8 Hz), 3.97 (s, 3H), 3.94 (s, 3H), 3.85 (s, 3H); ^13^C-NMR (100 MHz, CDCl_3_) δ 179.7, 160.4, 159.4, 155.3, 153.8, 136.2, 135.4, 131.7, 129.6, 127.1, 114.1, 110.4, 98.9, 67.4, 61.5, 61.4, 55.3; HRMS (EI): mass calculated for C_19_H_18_O_6_ [M^+^], 342.1103; found, 342.1106.

*(E)-5,7-Dihydroxy-6-methoxy-3-(4-methoxybenzylidene)chroman-4-one* (**8**). To a solution of the 3-(4-methoxybenzylidene)-4-chromanone (**19**) (15 mg, 0.04 mmol) in CH_2_Cl_2_ (5 mL) was added TMSI (13 μL, 0.09 mmol) at 0 °C for 1 h. The mixture was concentrated in vacuo. The residue was purified by flash column chromatography on silica gel (ethyl acetate:*n*-hexane = 1:3) to afford the resulting 5,7-dihydroxy-6-methoxy-4-chromanone (**8**) (9.1 mg, 63%). ^1^H-NMR (600 MHz, CDCl_3_) δ 7.80 (s, 1H), 7.27 (d, 2H, *J* = 7.2 Hz), 6.97 (d, 2H, *J* = 8.4 Hz), 6.03 (s, 1H), 5.28 (d, 2H, *J* = 1.8 Hz), 3.95 (s, 3H), 3.86 (s, 3H); ^13^C-NMR (100 MHz, CDCl_3_) δ 185.9, 160.8, 157.8, 157.2, 155.2, 137.2, 132.0, 128.4, 127.3, 126.7, 114.2, 103.3, 94.0, 67.4, 60.9, 55.4; HRMS (EI): mass calculated for C_18_H_16_O_6_ [M^+^], 328.0947; found, 328.0945.

## 4. Conclusions

In summary, we have successfully demonstrated the first total synthesis of naturally occurring homoisoflavonoids containing either a 7-hydroxy-5,6-dimethoxy or 5,7-dihydroxy-6-methoxy group on the A ring in four or five steps from the commercially available **11**. The key features of this synthetic route involve the following: (1) LiAlH_4_-mediated selective 1,4-reduction of 4-chromenone; (2) the aldol condensation of arylaldehydes, followed by catalytic hydrogenation; (3) TMSI-mediated C5-demethylation; (4) the selective debenzylation of 3-benzylidene-4-chromanone. These studies provide a timely contribution to the development of a practical synthetic approach to a variety of homoisoflavonoid analogues. Extension of this approach in the synthesis of various natural products and analogues and establishment of structure-activity relationship (SAR) profiles for 5,6,7-trisubstituted homoisoflavonoids compared to antiangiogenic cremastranone are currently underway.

## Figures and Tables

**Table 1:**
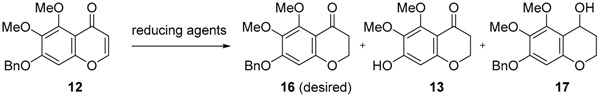
Reduction conditions of 7-benzyloxy-5,6-dimethoxy-4-chromenone (**12**).

Entry	Condition	Yield (%) ^a^		
		16	13	17
1	H_2_, Pd/C, MeOH, rt	- ^b^	99	-
2	NaBH_4_ (1.5 eq), EtOH, rt	-	-	<10
2	NaBH_4_ (2.5~5.0 eq), EtOH, rt	-	-	50
3	DIBAL ^c^ (1.0 eq), THF:toluene (1:1), −60 °C	20	-	20
4	LiAlH_4_ (2.0 eq), THF:Et_2_O (1:1), −60 °C	99	-	-

^a^ Isolated yield; ^b^ not found; ^c^ diisobutylaluminum hydride.

**Table 2:**
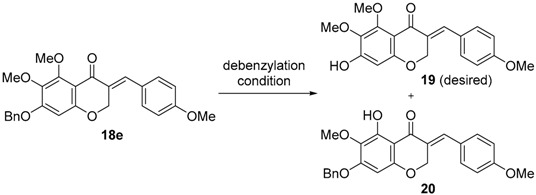
C7-debenzylation conditions of 5,6,7-trisubstituted 3-benzylidene-4-chromanone (**18e**).

Entry	Condition	Yield (%) ^a^	
		19	20
1	H_2_, Pd/C (3 mol %), MeOH, rt	65	-
2	TMSI ^b^, CH_2_Cl_2_, rt	-	60
3	HBr (47%), 50 °C	-	60
4	TiCl_4_ (1 eq), CH_2_Cl_2_, 0 °C	NR ^c^	
5	Li, naphthalene, THF, −25 °C	NR	

^a^ Isolated yield; ^b^ trimethylsilyl iodide; ^c^ no reaction.

## Supplementary Materials

Supplementary materials can be accessed at: http://www.mdpi.com/1420-3049/21/8/1058/s1.
