# Supplementary material for: Synthesis of Natural Homoisoflavonoids Having Either 5,7-Dihydroxy-6-methoxy or 7-Hydroxy-5,6-dimethoxy Groups
Source: Molecules. 2016 Aug 13;21(8):1058. doi: 10.3390/molecules21081058 (PMC5139345; doi:10.3390/molecules21081058)
Supplement: Supplementary file 1 [file molecules-21-01058-s001.pdf]

Supplementary Materials: Synthesis of Natural Homoisoflavonoids Having Either 5,7-Dihydroxy-6-methoxy or 7-Hydroxy-5,6-dimethoxy Groups

Hyungjun Lee, Yue Yuan, Inmoo Rhee, Timothy W. Corson and Seung-Yong Seo

Table S1. NMR comparison of natural and synthetic homoisoflavonoids (**2**~**8**).

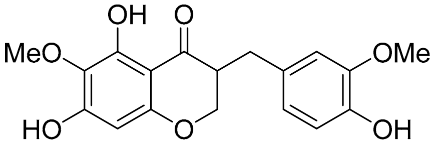

2

Site	^{1}H-NMR of Lit. 2	^{1}H-NMR of Synthetic 2	Δ(δL − δS)	^{13}C-NMR of Lit. 2	^{13}C-NMR of Synthetic 2	Δ(δL − δS)
	CD₃OD	CD₃OD		CD₃OD	CD₃OD	
2H-1	4.08	4.09	−0.01	199.5	199.9	−0.4
2H-2	4.24	4.25	−0.01	160.8	160.7	0.1
3H	2.83	2.86	−0.03	159.8	159.8	0
5H	12.22	12.2	0.02	156.7	156.6	0.1
6H	3.77	3.77	0	148.9	148.8	0.1
8H	5.91	5.9		146.1	146	0.1
7H	10.5	*		130.8	130.6	0.2
9H-1	2.66	2.68	−0.02	130.6	130.2	0.4
9H-2	3.1	3.12	−0.02	122.7	122.4	0.3
4′H	8.78	*		116.3	116	0.3
2′H	6.8	6.81	−0.01	113.7	113.3	0.4
3′H	3.83	3.83	0	103	102.6	0.4
5′H	6.73	6.73	0	95.8	95.6	0.2
6′H	6.65	6.67	−0.02	70.4	70.1	0.3
				61	60.7	0.3
				56.5	56.1	0.4
				33.5	33.2	0.3

(Lit.) *Phytochemistry* **1987**, *26*, 285–290. * not detected.

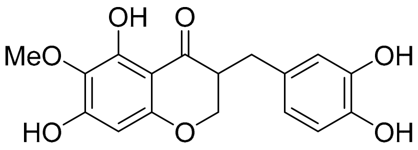

3

Site	^{1}H-NMR of Lit. 3	^{1}H-NMR of Synthetic 3	Δ(δL − δS)	^{13}C-NMR of Lit. 3	^{13}C-NMR of Synthetic 3	Δ(δL − δS)
	CD3OD	CD3OD		CD3OD	CD3OD	
2H-1	4.08	4.08	0	200.1	200.2	−0.1
2H-2	4.25	4.24	0.01	160.9	160.7	0.2
3H	2.79	2.8	−0.01	160.1	160.1	0
5H	12.22	12.2	0.02	156.8	156.9	−0.1
6H	3.8	3.77	0.03	146.4	146.4	0
8H	5.92	5.91	0.01	145.1	145.1	0
7H	10.66	*		130.9	130.8	0.1
9H-1	2.6	2.6	0	130.5	130.4	0.1
9H-2	3.06	3.05	0.01	121.5	121.4	0.1
4′H	8.72	*		117.1	117.1	0
2′H	6.7	6.67	0.03	116.5	116.4	0.1
3′H	8.79	*		102.9	103	−0.1
5′H	6.73	6.71	0.02	95.8	95.8	0
6′H	6.56	6.55	0.01	70.2	70.3	−0.1
				61	61	0
				33.2	33.2	0

(Lit.) *Phytochemistry* **1987**, *26*, 285–290. * not detected.

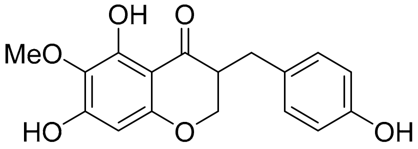

4

Site	^{1}H-NMR of Lit. 4	^{1}H-NMR of Synthetic 4	Δ(δL − δS)	^{13}C-NMR of Lit. 4	^{13}C-NMR of Synthetic 4	Δ(δL − δS)
	CD3OD	CD3OD		CD3OD	CD3OD	
2H-1	4	4	0	199.9	199.9	0
2H-2	4.17	4.2	−0.03	161.1	160.5	0.6
3H	2.75	2.8	−0.05	160	159.8	0.2
5H				157.1	156.9	0.2
6H	3.76	3.7	0.06	156.7	156.5	0.2
8H	5.89	5.9	−0.01	131	130.8	0.2
7H				130.4	130.1	0.3
9H-1	2.59	2.6	−0.01	130	129.8	0.2
9H-2	3.07	3.1	−0.03	116.3	116.1	0.2
4′H				102.7	102.6	0.1
2′H	7.02	7	0.02	95.9	95.5	0.4
3′H				70.2	69.9	0.3
5′H	6.72	6.7	0.02	60.9	60.6	0.3
6′H				49.1	47.8	1.3
				32.9	32.7	0.2

(Lit.) *Phytochemistry* **1987**, *26*, 285–290.

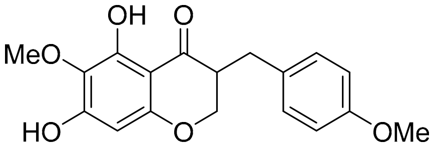

5

Site	¹H-NMR of Lit. 5	¹H-NMR of Synthetic 5	Δ(δL − δS)	¹³C-NMR of Lit. 5	¹³C-NMR of Synthetic 5	Δ(δL − δS)
	CD3OD	CD3OD		CD3OD	CD3OD	
2H-1	4.1	4.06	0.04	200.1	200.1	0
2H-2	4.27	4.23	0.04	160.7	160.7	0
3H	2.85	2.84	0.01	160.1	160.1	0
5H				160	160	0
6H	3.81	3.76	0.05	156.5	156.9	-0.4
8H	5.95	5.9	0.05	131.4	131.4	0
7H				131.4	131.2	0.2
9H-1	2.73	2.7	0.03	131.2	130.4	0.8
9H-2	3.26	3.13	0.13	115.1	115.1	0
4′H				103	103	0
2′H	7.19	7.15	0.04	95.8	95.8	0
3′H	3.81	3.77	0.04	70.3	70.3	0
5′H	6.91	6.86	0.05	61	61	0
6′H				55.7	55.7	0
				32.9	32.9	0

(Lit.) *Phytochemistry* **1999**, *51*, 943–946.

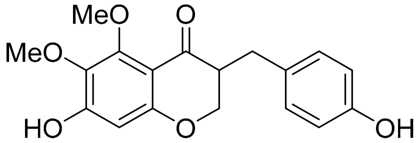

6

Site	¹H-NMR of Lit. 6	¹H-NMR of Synthetic 6	Δ(δL − δS)	¹³C-NMR of Lit. 6	¹³C-NMR of Synthetic 6	Δ(δL − δS)
	CDCl3	CDCl3		CDCl3	CDCl3	
2H-1	4.04	4.0	0.04	191.5	191.7	−0.2
2H-2	4.23	4.2	0.03	159.8	159.8	0
3H	2.68	2.7	−0.02	155.5	155.6	−0.1
5H	3.9	3.9		154.1	154.3	−0.2
6H	3.9	3.9		153.4	153.5	−0.1
8H	6.28	6.3	−0.02	135.2	135.2	0
7H				130.5	130.3	0.2
9H-1	2.62	2.6	0.02	130.3	130.3	0
9H-2	3.13	3.1	0.03	115.4	115.4	0
4′H				108.7	108.6	0.1
2′H	7.07	7.0	0.07	98.8	98.8	0
3′H	6.75	6.7	0.05	68.8	68.8	0
5′H	6.75	6.7	0.05	61.5	61.5	0
6′H	7.07	7.0	0.07	61.4	61.4	0
				48.5	48.5	0
				31.9	32	−0.1

(Lit.) *Biochemical Systematics and Ecology* **2006**, *34*, 114–118.

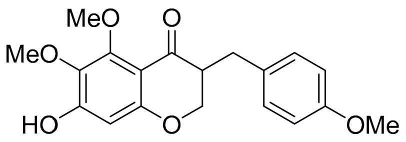

7

Site	^{1}H-NMR of Lit. 7	^{1}H-NMR of Synthetic 7	Δ(δL − δS)	^{13}C-NMR of Lit. 7	^{13}C-NMR of Synthetic 7	Δ(δL − δS)
	CD_3COCD_3	CD_3COCD_3		CD_3COCD_3	CD_3COCD_3	
2H-1	4.15	4.14	0.01	190	190	0
2H-2	3.95	3.94	0.01	159.5	159.6	−0.1
3H	2.62	2.62	0	158.4	158.5	−0.1
5H				157.6	157.1	0.5
6H				154.6	154.7	−0.1
8H	6.09	6.09	0	136.4	136.3	0.1
7H				130.7	130.8	−0.1
9H-1	3.01	3	0.01	130	130.1	−0.1
9H-2	2.59	2.58	0.01	113.8	113.9	−0.1
4′H	3.64	3.64	0	108	108.5	−0.5
2′H	7.07	7.07	0	99.1	99.1	0
3′H	6.76	6.75	0.01	68.9	69.1	−0.2
5′H	3.69	3.71	−0.02	60.7	60.8	−0.1
6′H	3.63	3.64	−0.01	60.4	60.6	−0.2
				54.5	54.6	−0.1
				48.2	48.3	−0.1
				31.5	31.5	0

(Lit.) *Phytochemistry Letters* **2012**, *5*, 591–595.

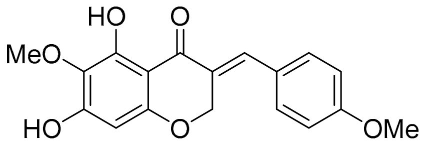

8

Site	^{1}H-NMR of Lit. 8	^{1}H-NMR of Synthetic 8	Δ(δL − δS)	^{13}C-NMR of Lit. 8	^{13}C-NMR of Synthetic 8	Δ(δL − δS)
	$CDCl_3$	$CDCl_3$		$CDCl_3$	$CDCl_3$	
2H-1	5.28	5.28	0	186.2	186.2	0
2H-2	5.28	5.28		161.1	161.1	0
3H				158.1	158.1	0
5H				157.5	157.5	0
6H	3.94	3.95	−0.01	155.5	155.5	0
8H	6.03	6.03	0	137.5	137.5	0
7H				132.3	132.3	0
9H-1	7.8	7.8	0	128.7	128.7	0
9H-2				127.6	127.7	−0.1
4′H	3.86	3.86	0	126.9	127	−0.1
2′H	7.25	7.27	−0.02	114.5	114.6	−0.1
3′H	6.97	6.97	0	103.6	103.6	0
5′H				94.3	94.3	0
6′H				67.7	67.7	0
				61.2	61.2	0
				55.7	55.7	0

(Lit.) *Phytochemistry* **1999**, *52*, 947–955.

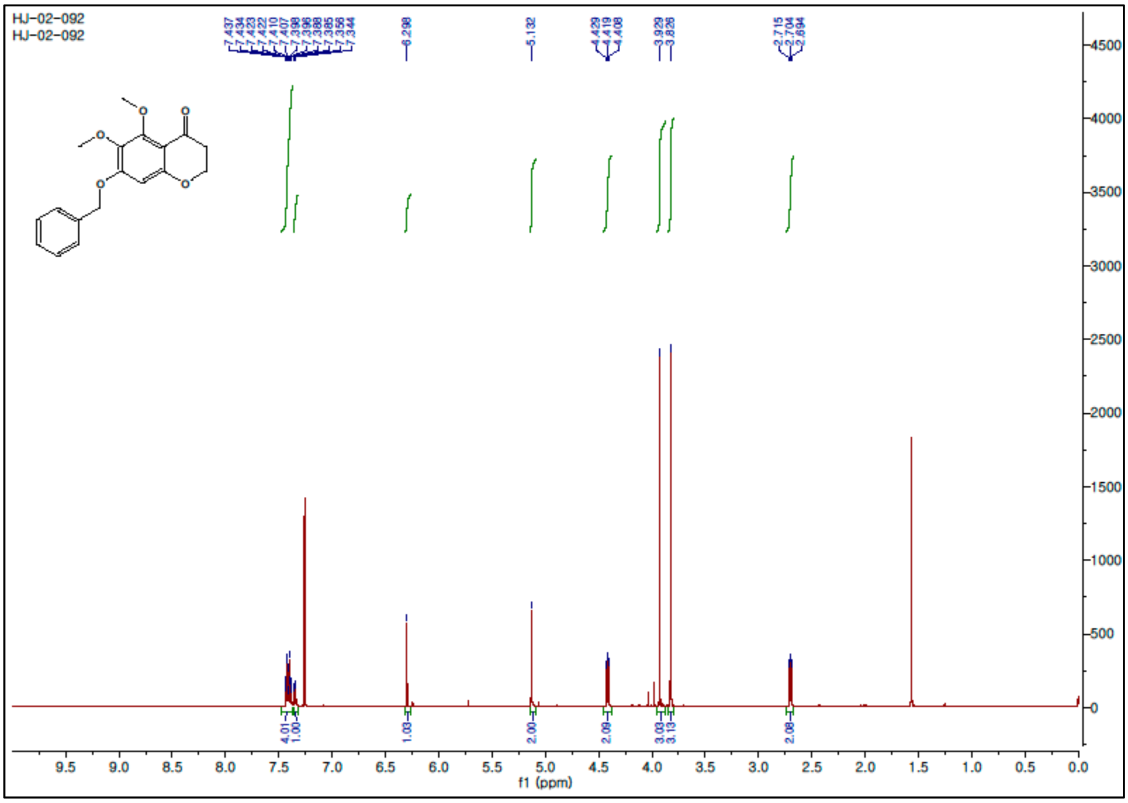

Figure S1. ^{1}H-NMR ($CDCl_3$, 600 MHz) of compound **16**.

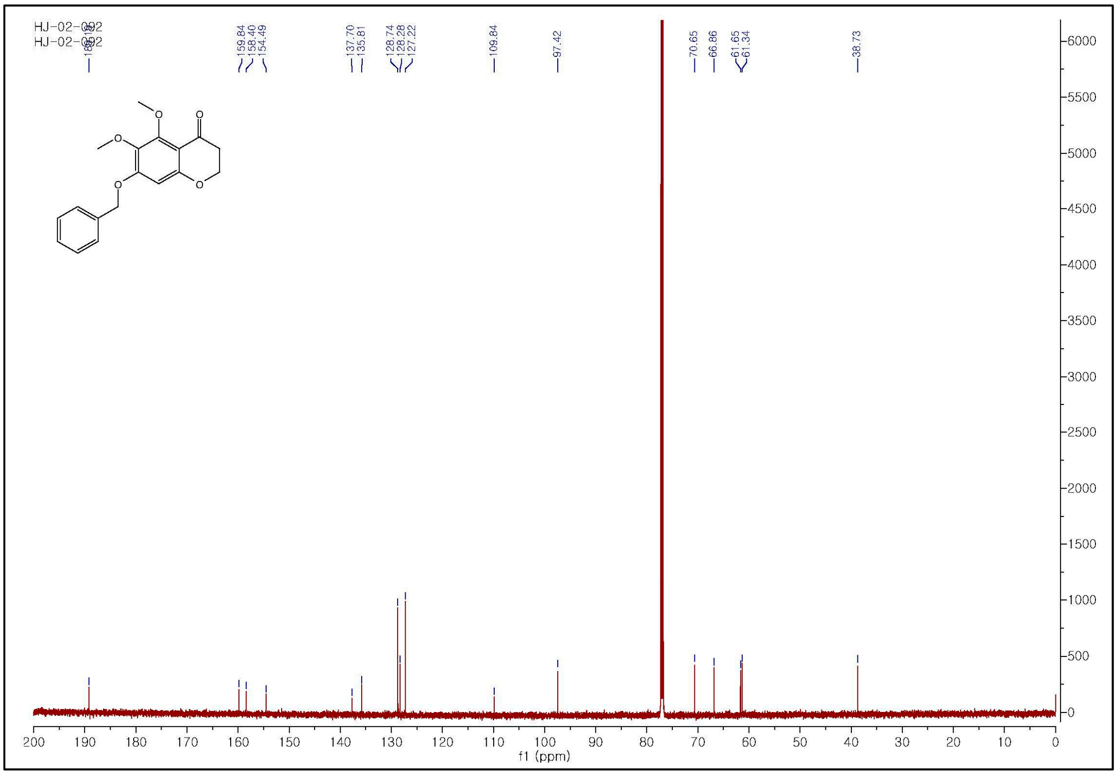

Figure S2. ^{13}C-NMR (150 MHz, $CDCl_3$) of compound **16**.

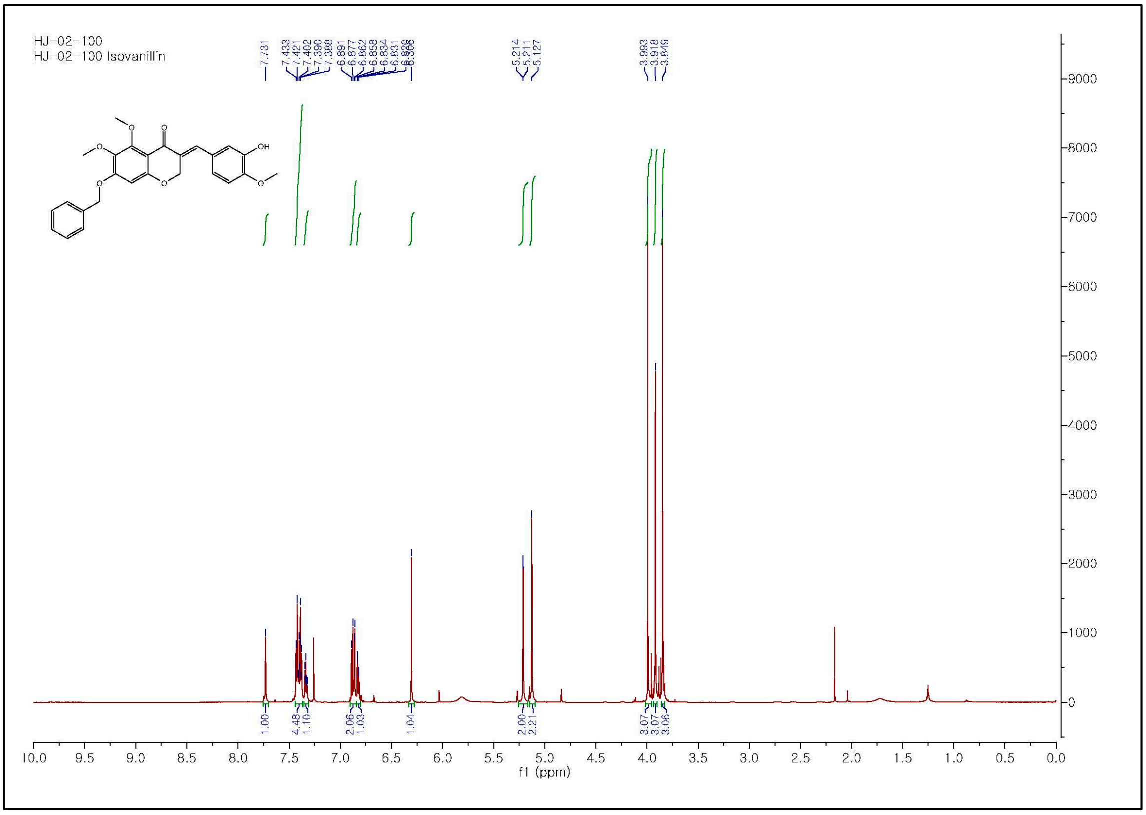

Figure S3. ^{1}H-NMR ($CDCl_3$, 600 MHz) of compound **18a**.

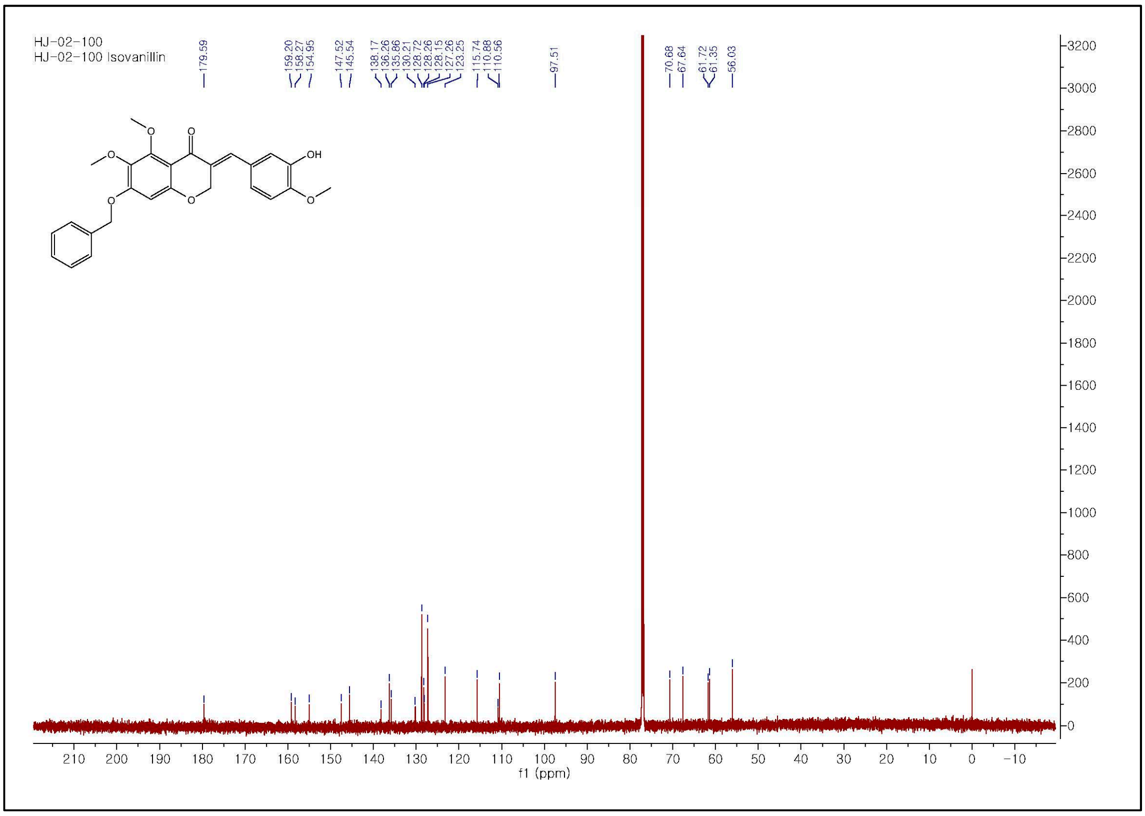

Figure S4. ^{13}C-NMR (150 MHz, $CDCl_3$) of compound **18a**.

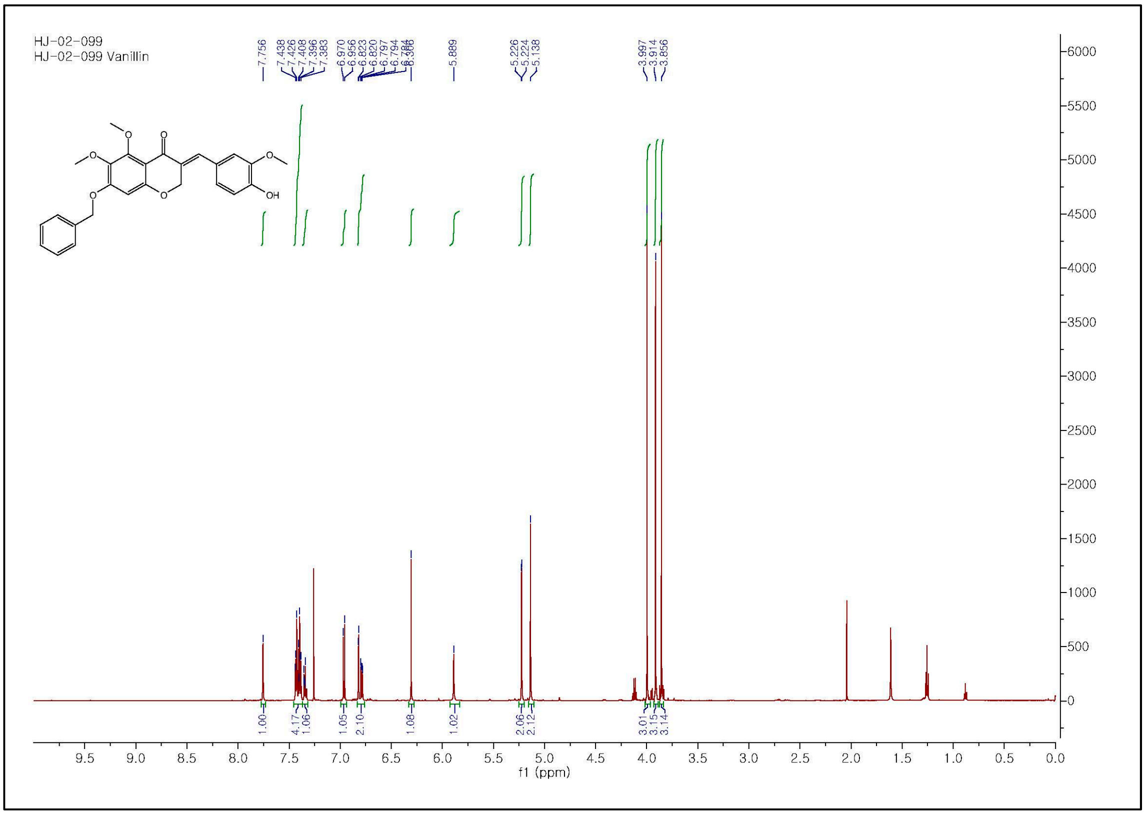

Figure S5. ^{1}H-NMR ($CDCl_3$, 600 MHz) of compound **18b**.

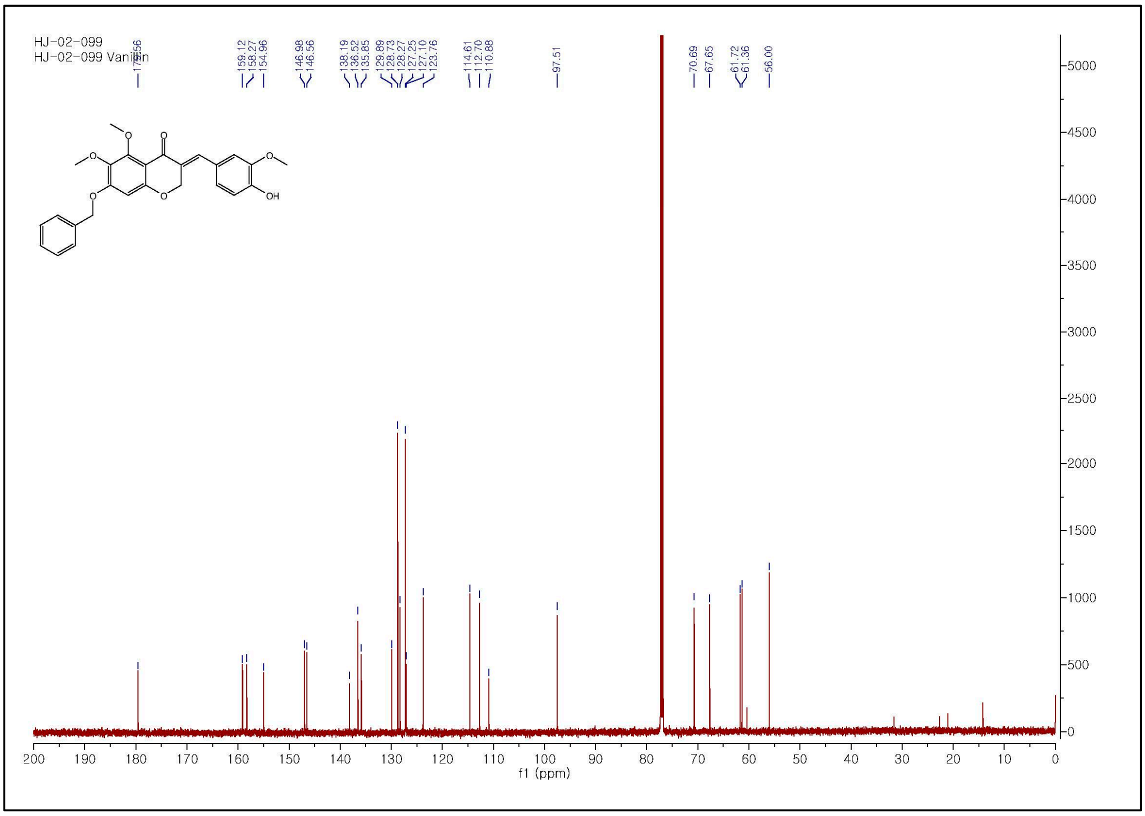

Figure S6. ^{13}C-NMR (150 MHz, $CDCl_3$) of compound **18b**.

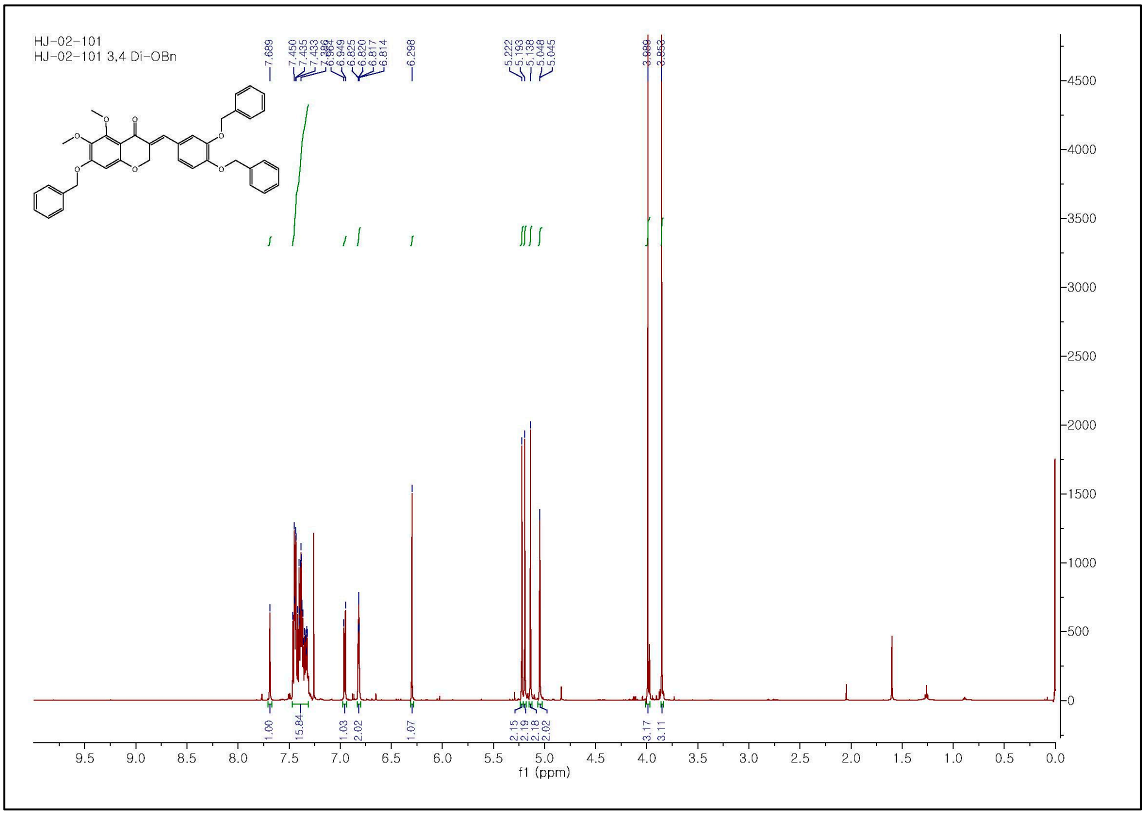

Figure S7. ^{1}H-NMR ($CDCl_3$, 600 MHz) of compound **18c**.

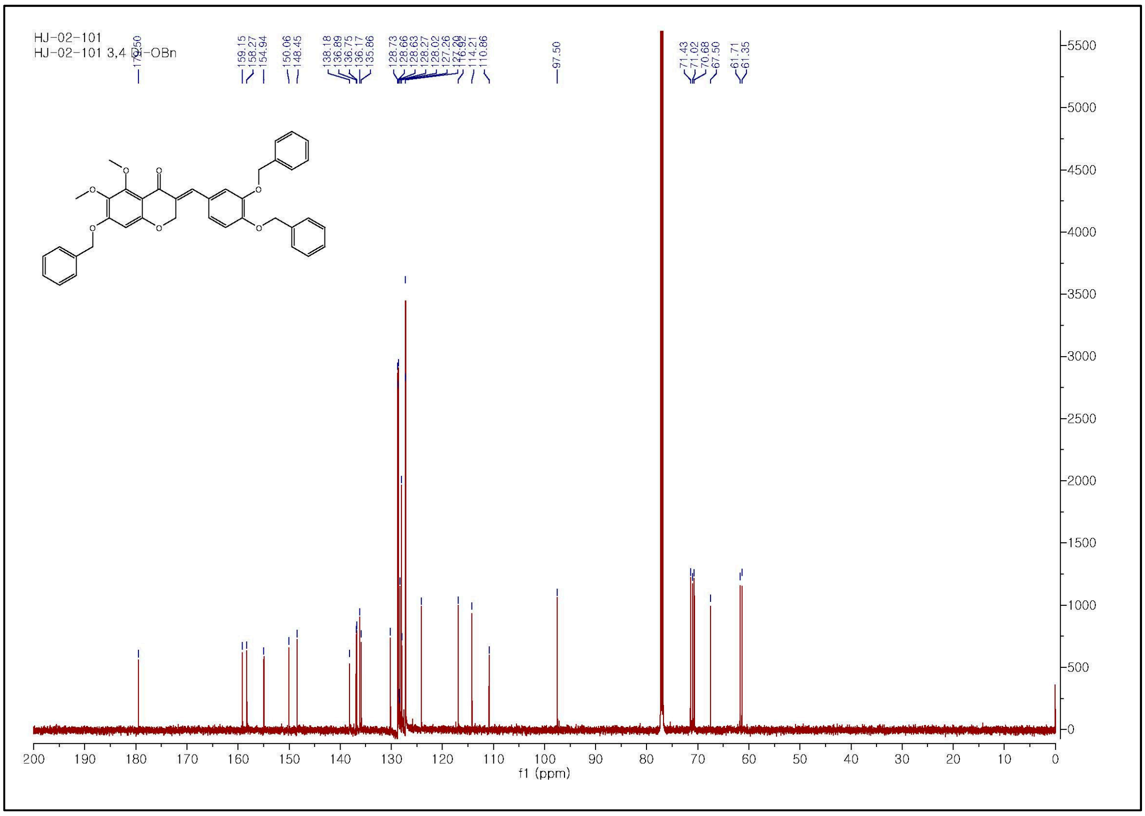

Figure S8. ^{13}C-NMR (150 MHz, $CDCl_3$) of compound **18c**.

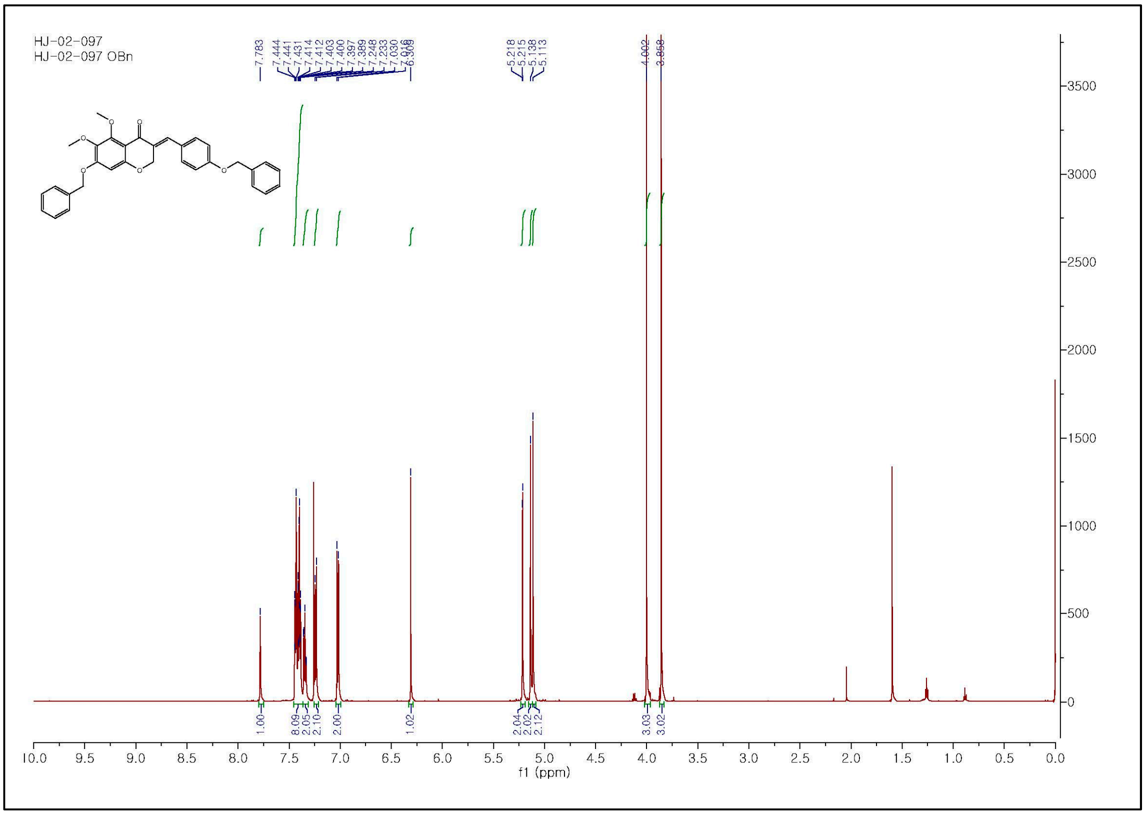

Figure S9. ^{1}H-NMR ($CDCl_3$, 600 MHz) of compound **18d**.

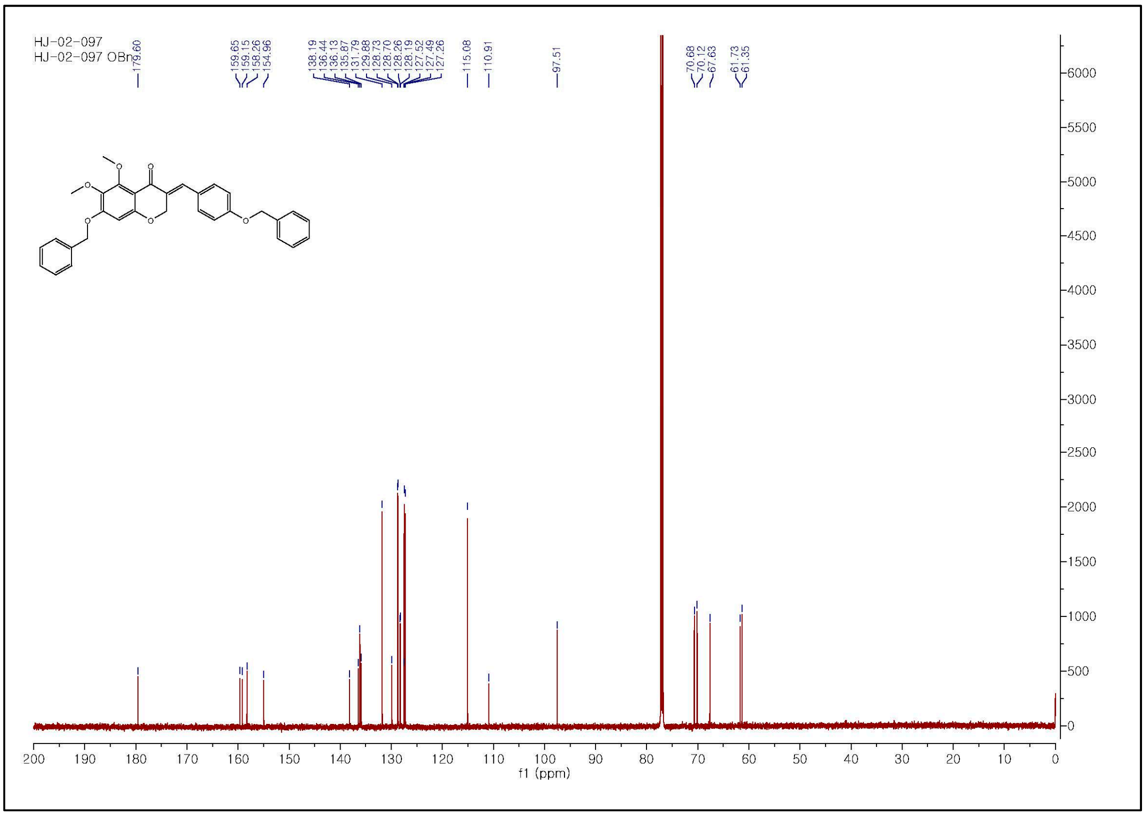

Figure S10. ^{13}C-NMR (150 MHz, $CDCl_3$) of compound **18d**.

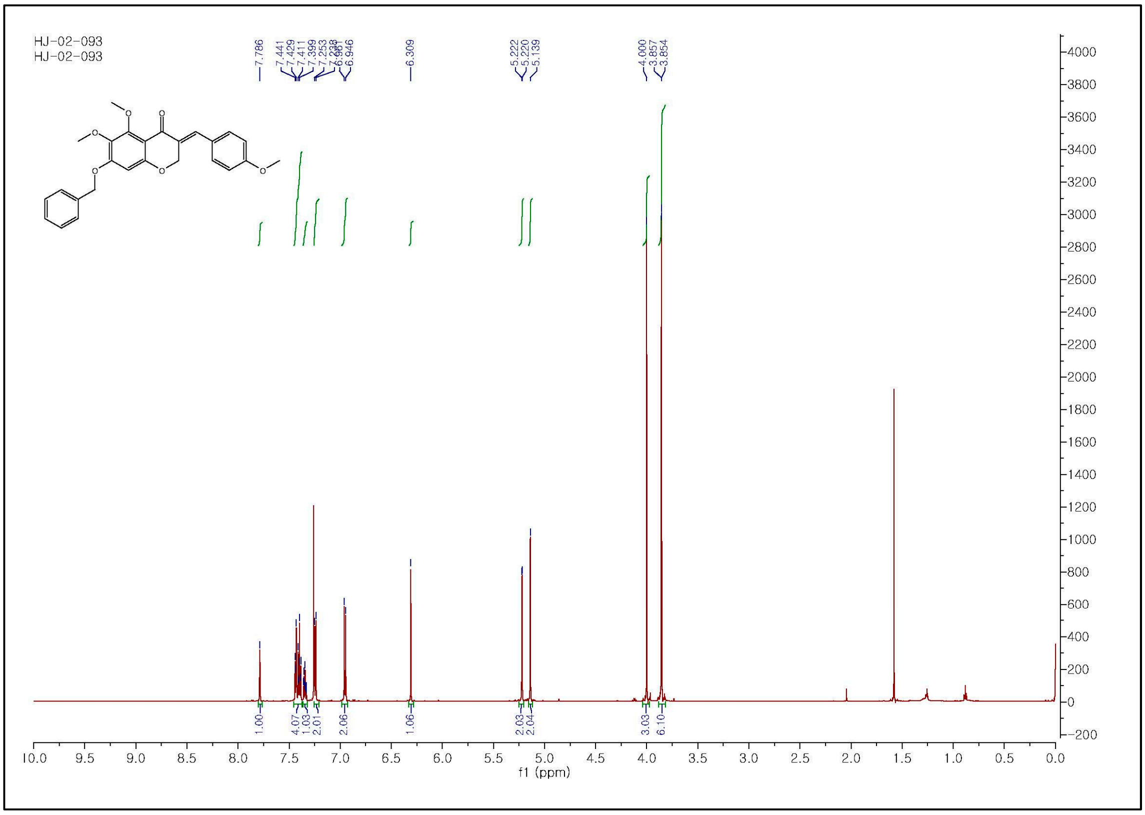

Figure S11. ^{1}H-NMR ($CDCl_3$, 600 MHz) of compound **18e**.

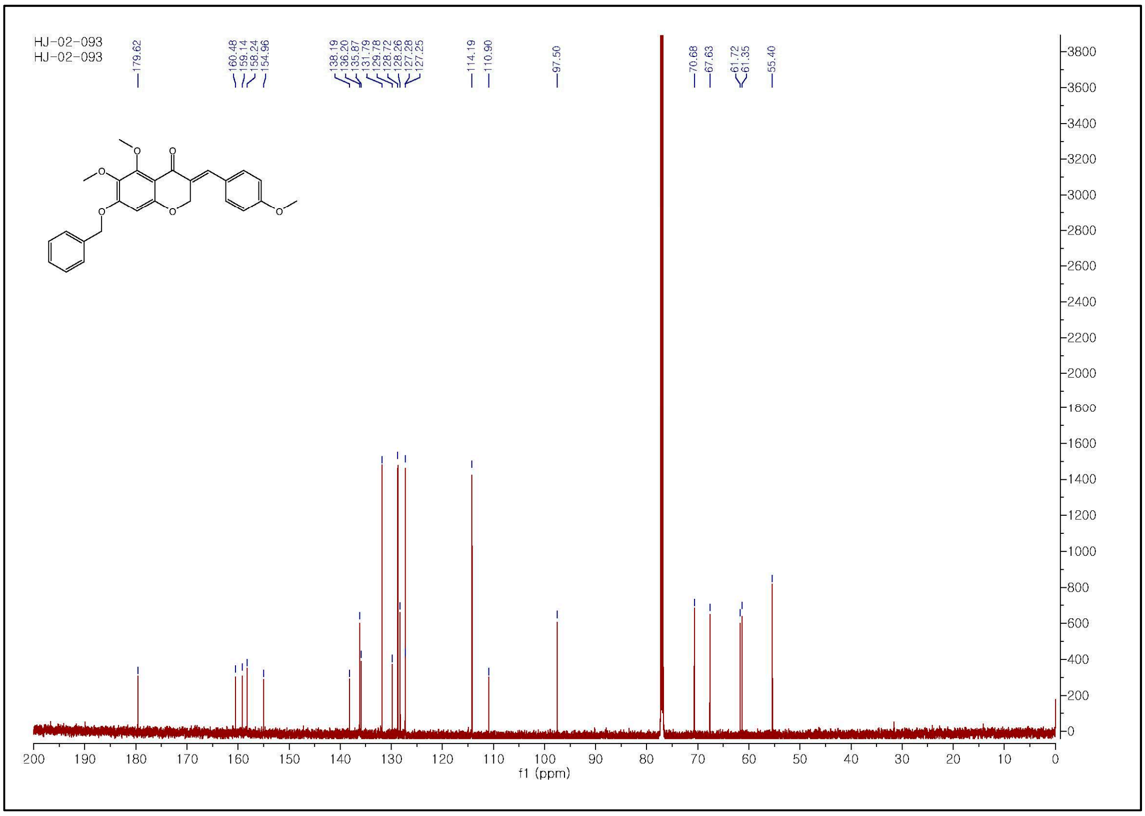

Figure S12. ^{13}C-NMR (150 MHz, $CDCl_3$) of compound **18e**.

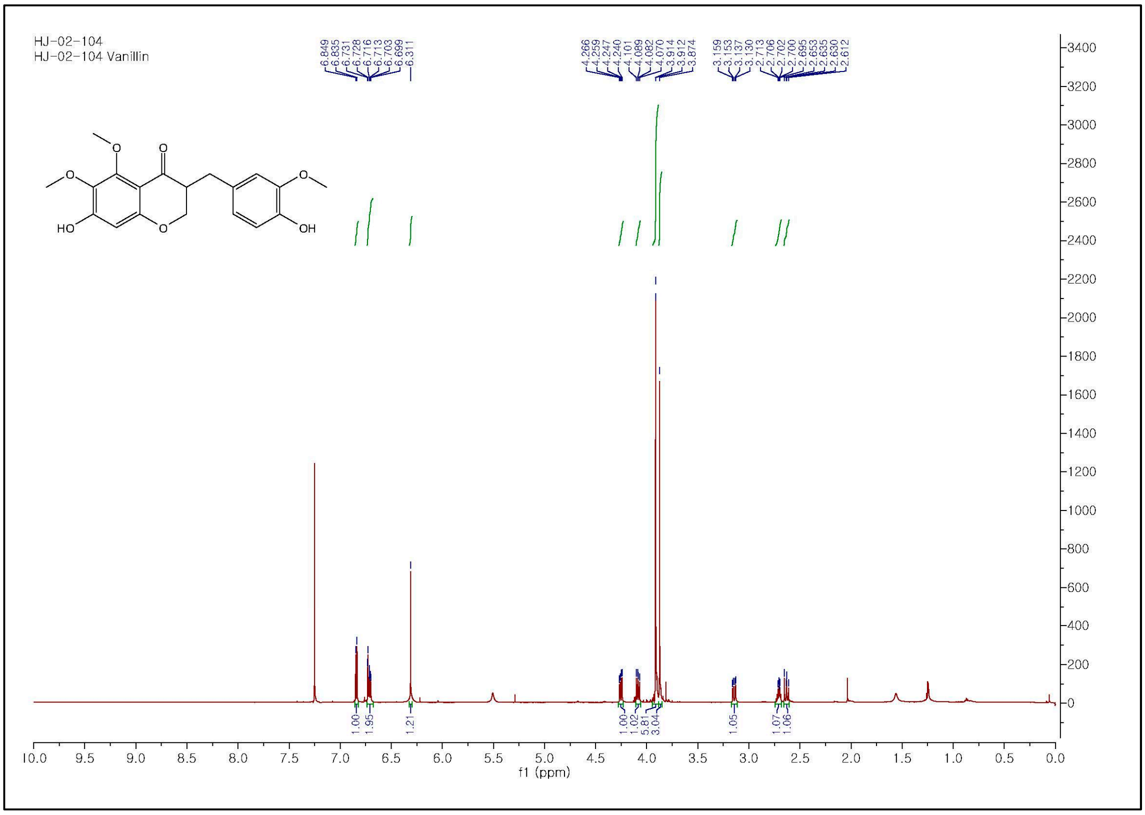

Figure S13. ^{1}H-NMR ($CDCl_3$, 600 MHz) of compound **15b**.

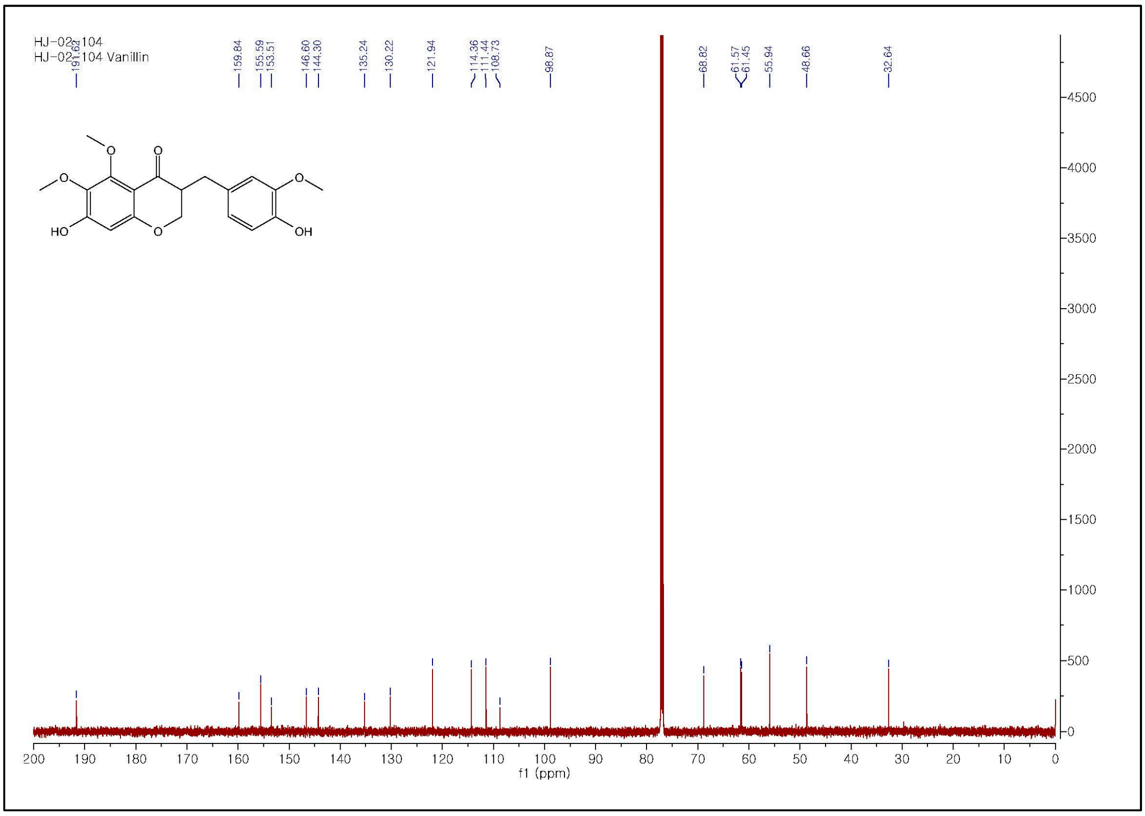

Figure S14. ^{13}C-NMR (150 MHz, $CDCl_3$) of compound **15b**.

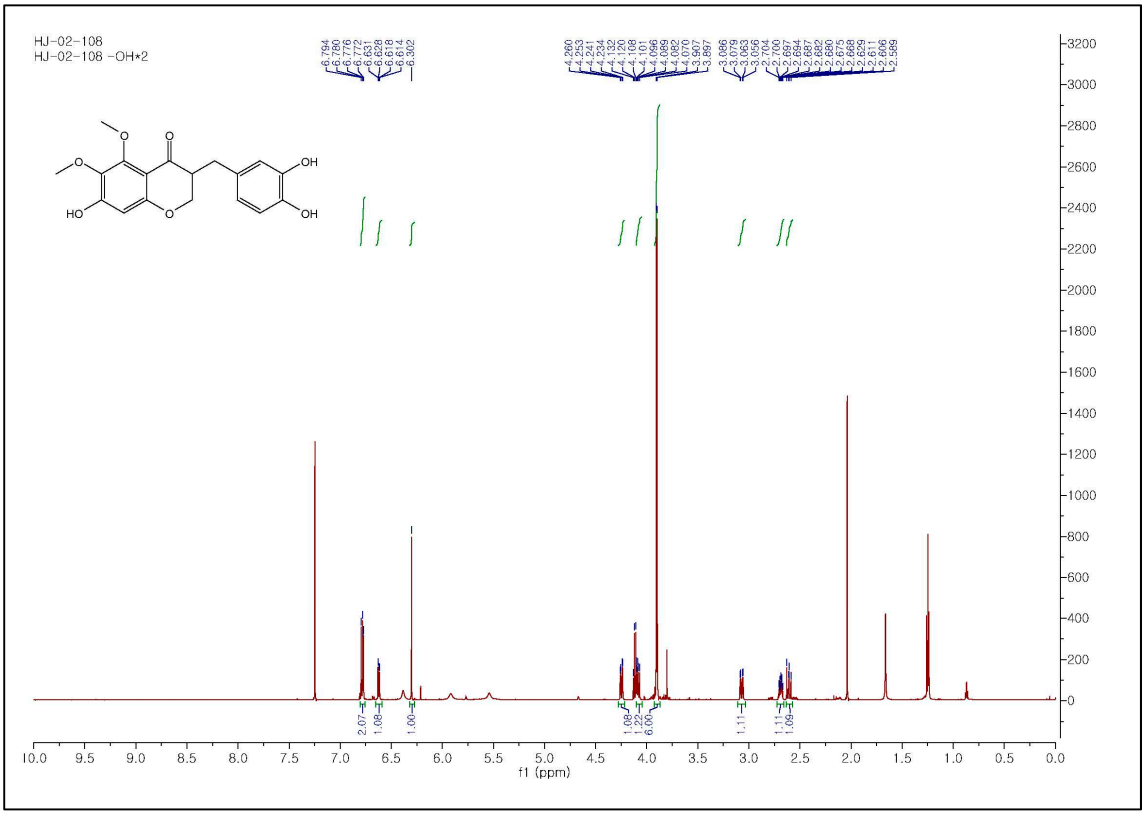

Figure S15. ^{1}H-NMR ($CDCl_3$, 600 MHz) of compound **15c**.

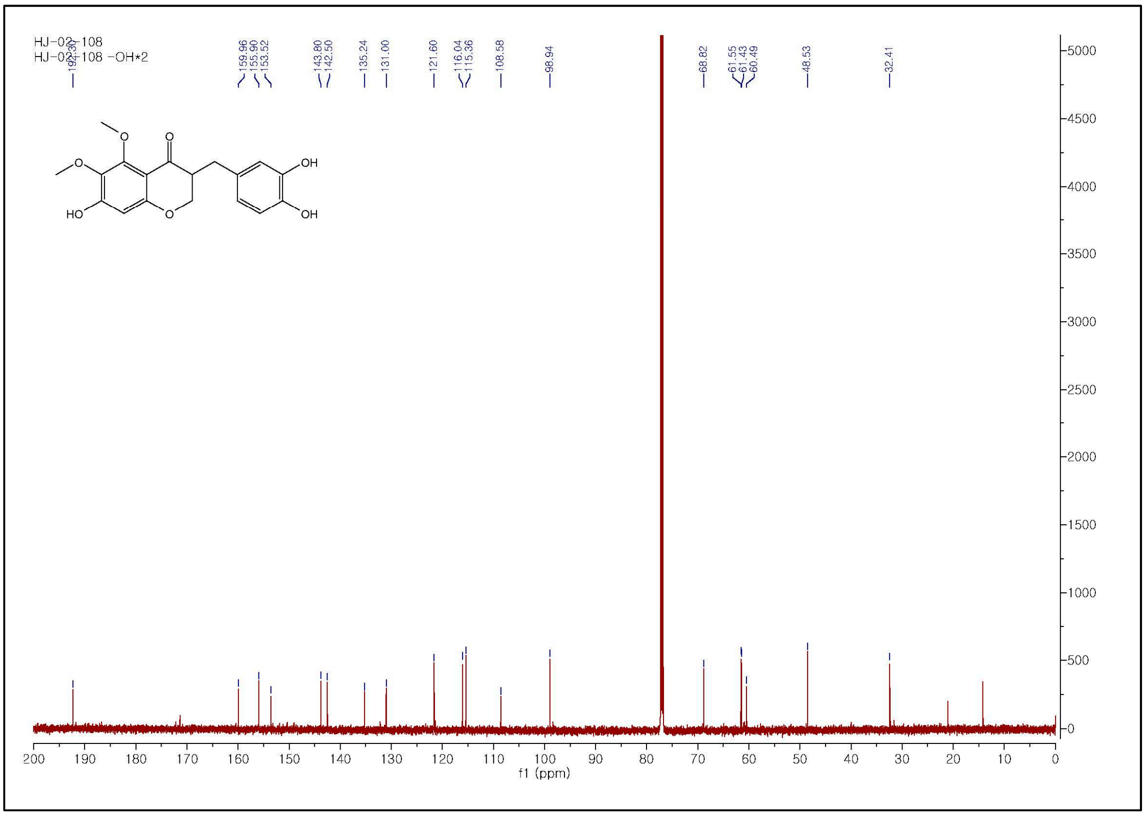

Figure S16. ^{13}C-NMR (150 MHz, $CDCl_3$) of compound **15c**.

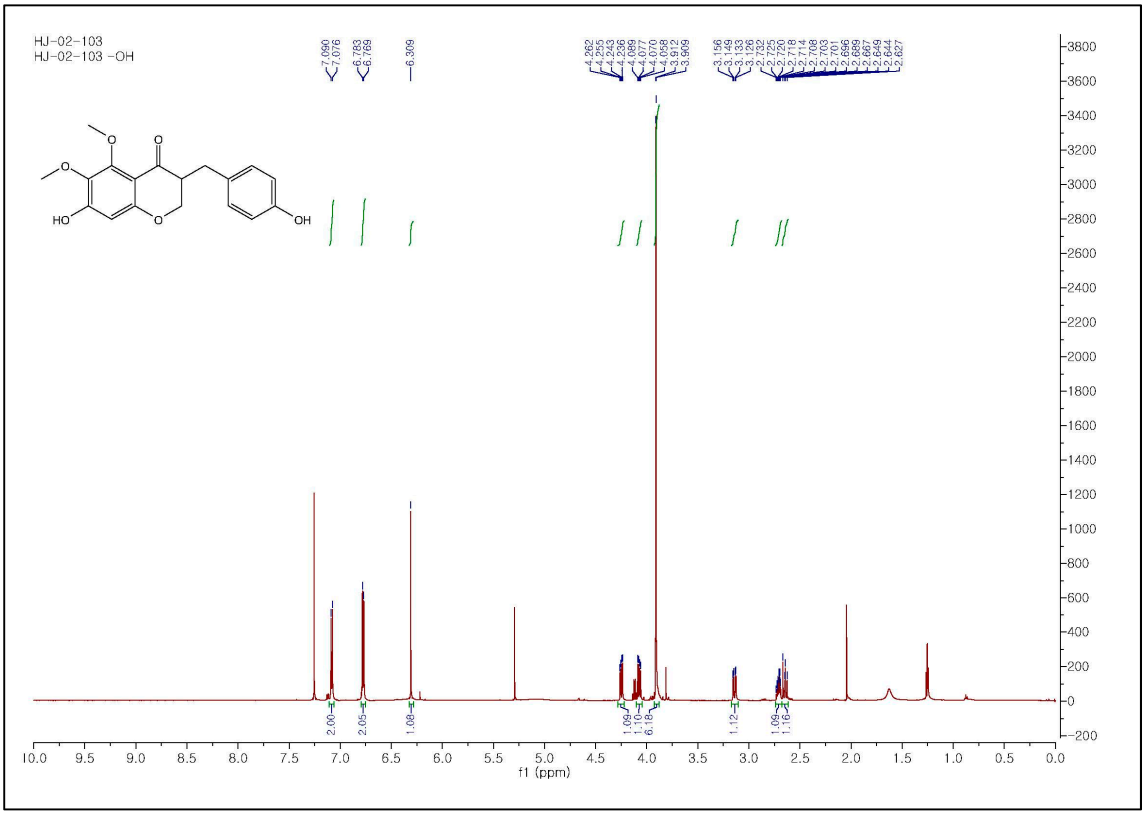

Figure S17. ^{1}H-NMR ($CDCl_3$, 600 MHz) of compound **6**.

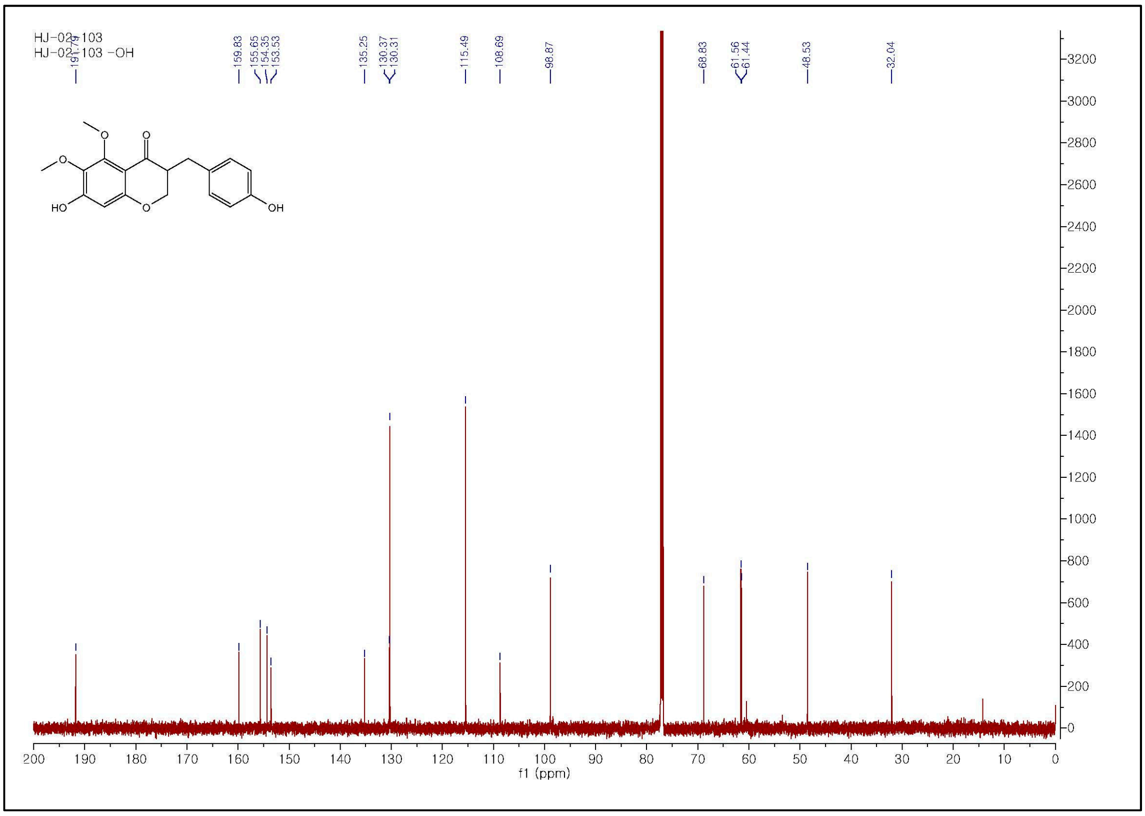

Figure S18. ^{13}C-NMR (150 MHz, $CDCl_3$) of compound **6**.

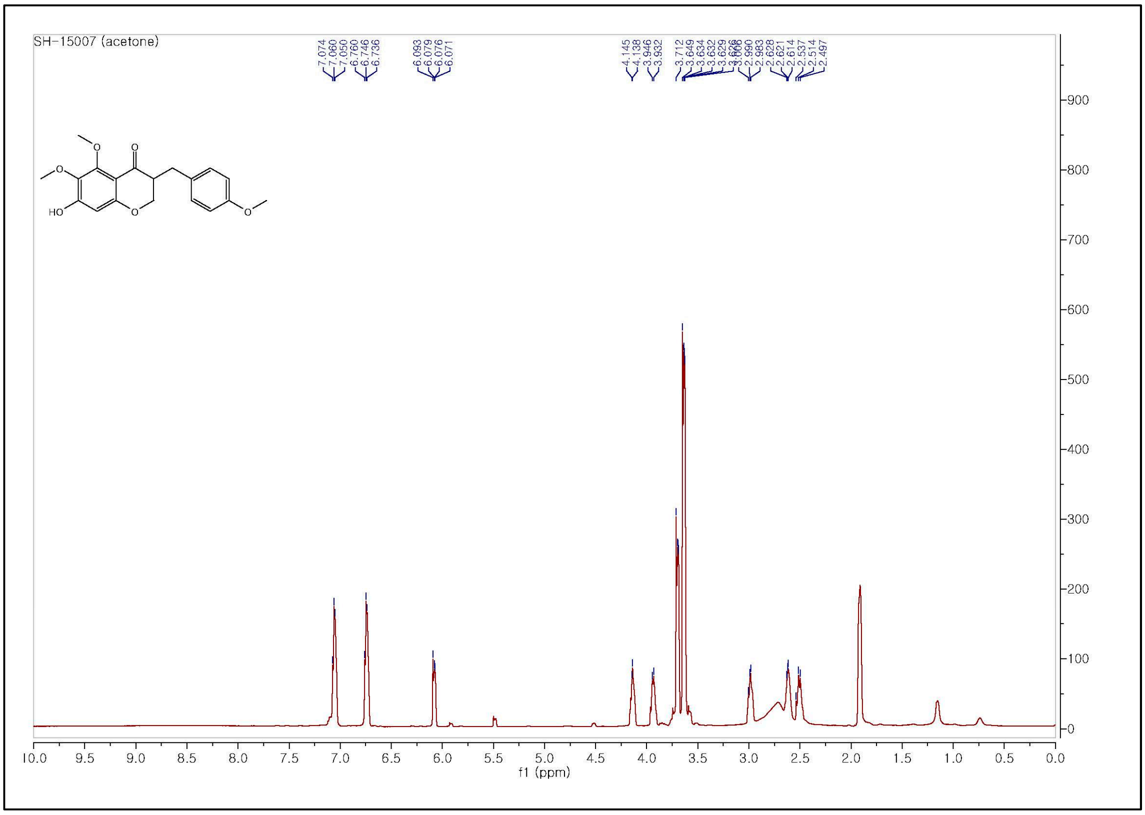

Figure S19. ^{1}H-NMR (600 MHz, CD_3COCD_3) of compound **7**.

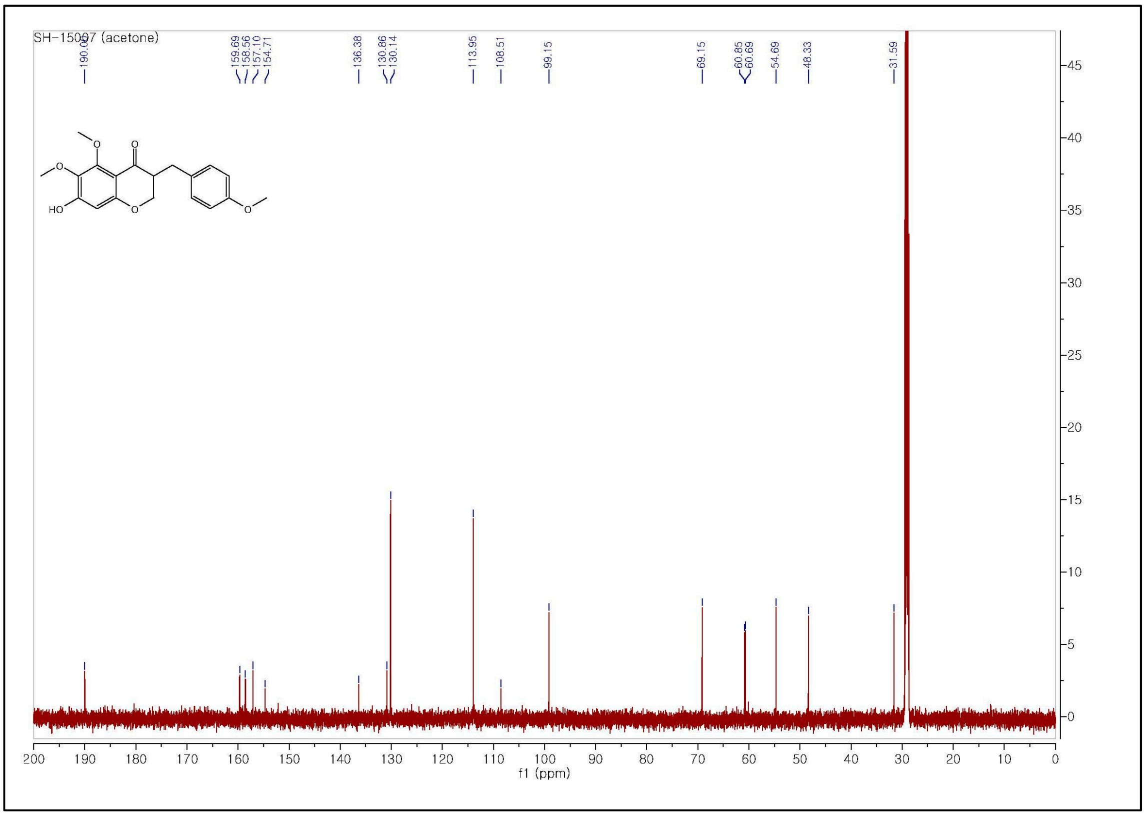

Figure S20. ^{13}C-NMR (150 MHz, $CDCl_3$) of compound **7**.

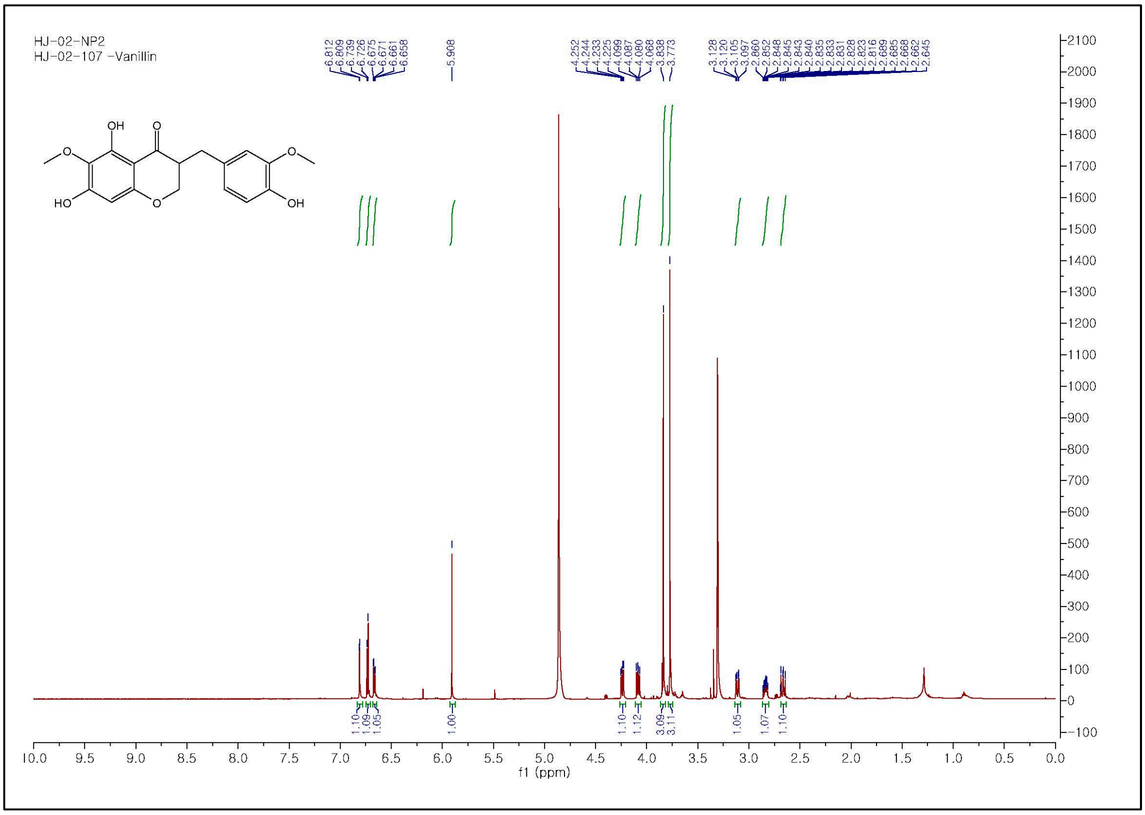

Figure S21. ^{1}H-NMR (600 MHz, CD_3OD) of compound **2**.

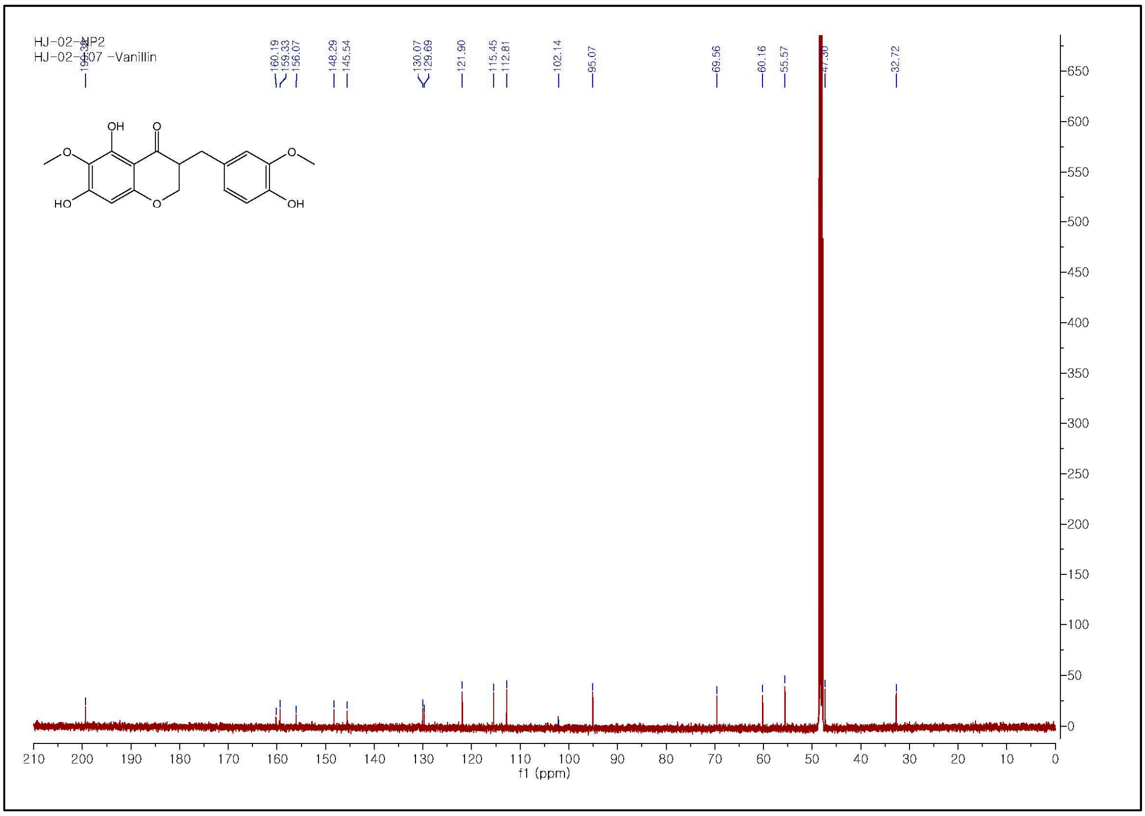

Figure S22. ^{13}C-NMR (150 MHz, CD_3OD) of compound **2**.

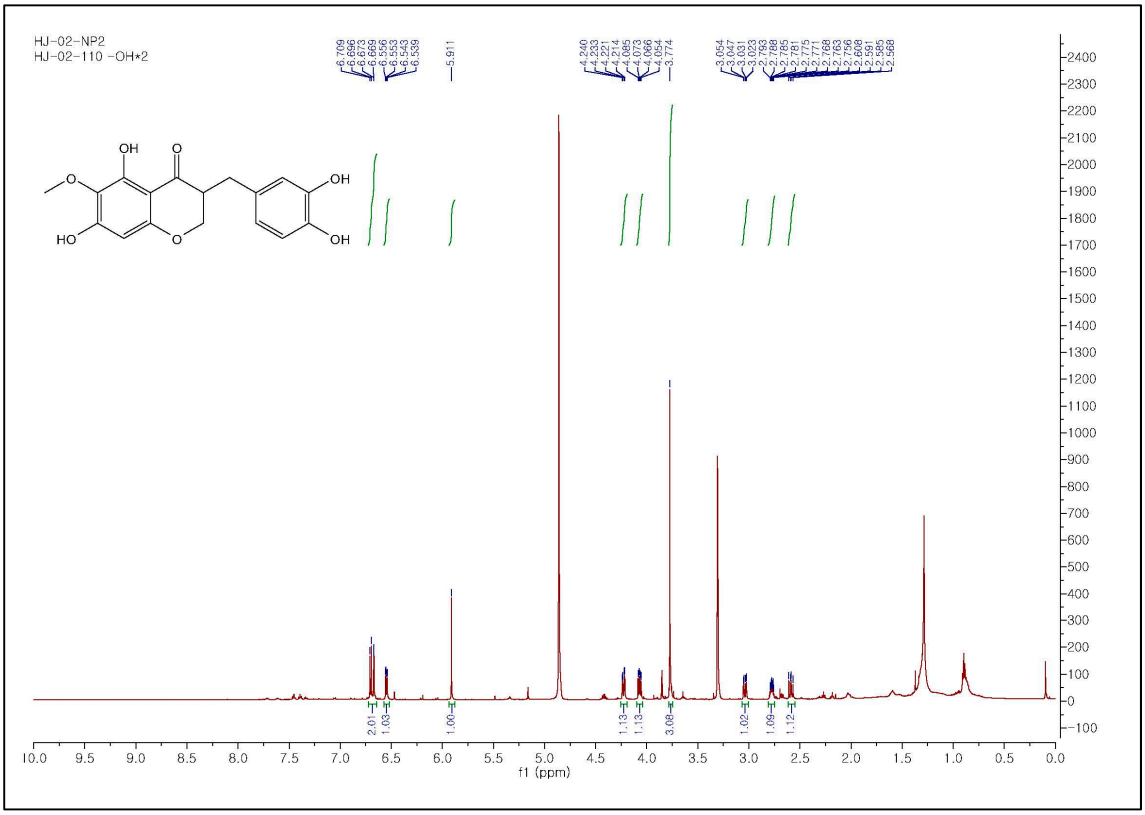

Figure S23. ^{1}H-NMR (600 MHz, CD_3OD) of compound **3**.

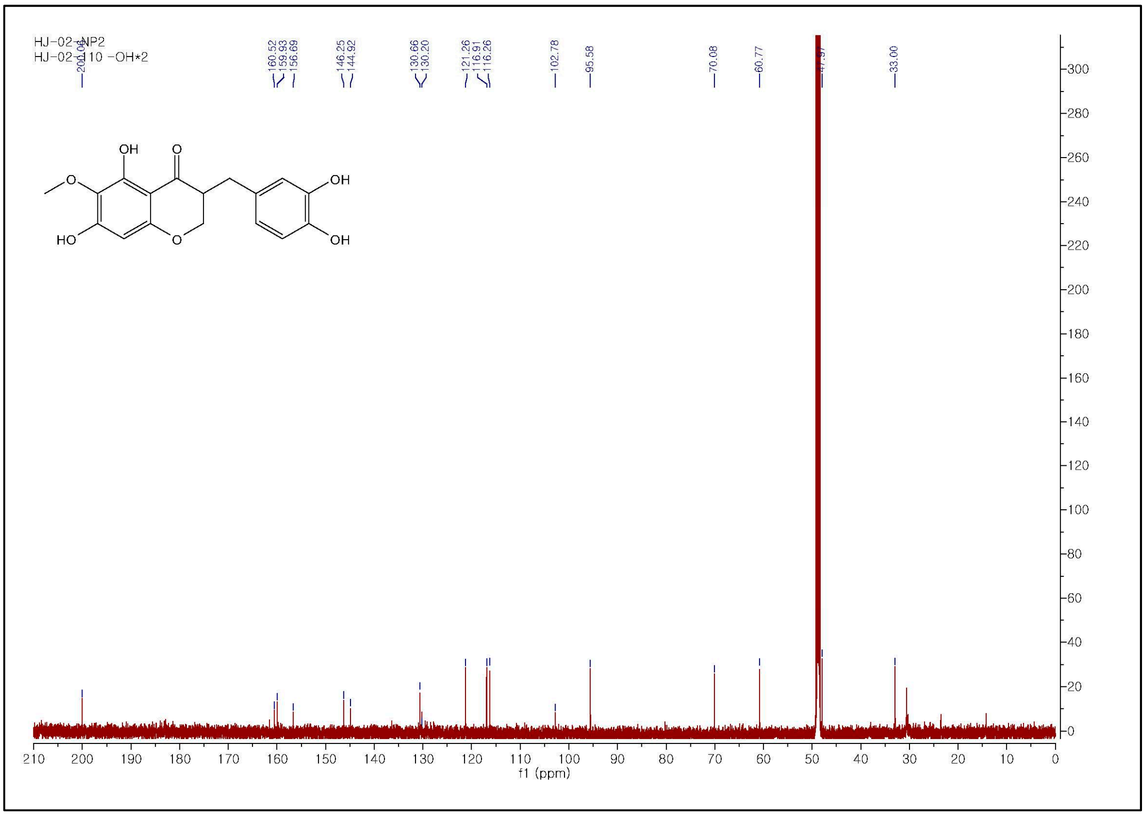

Figure S24. ^{13}C-NMR (150 MHz, CD_3OD) of compound **3**.

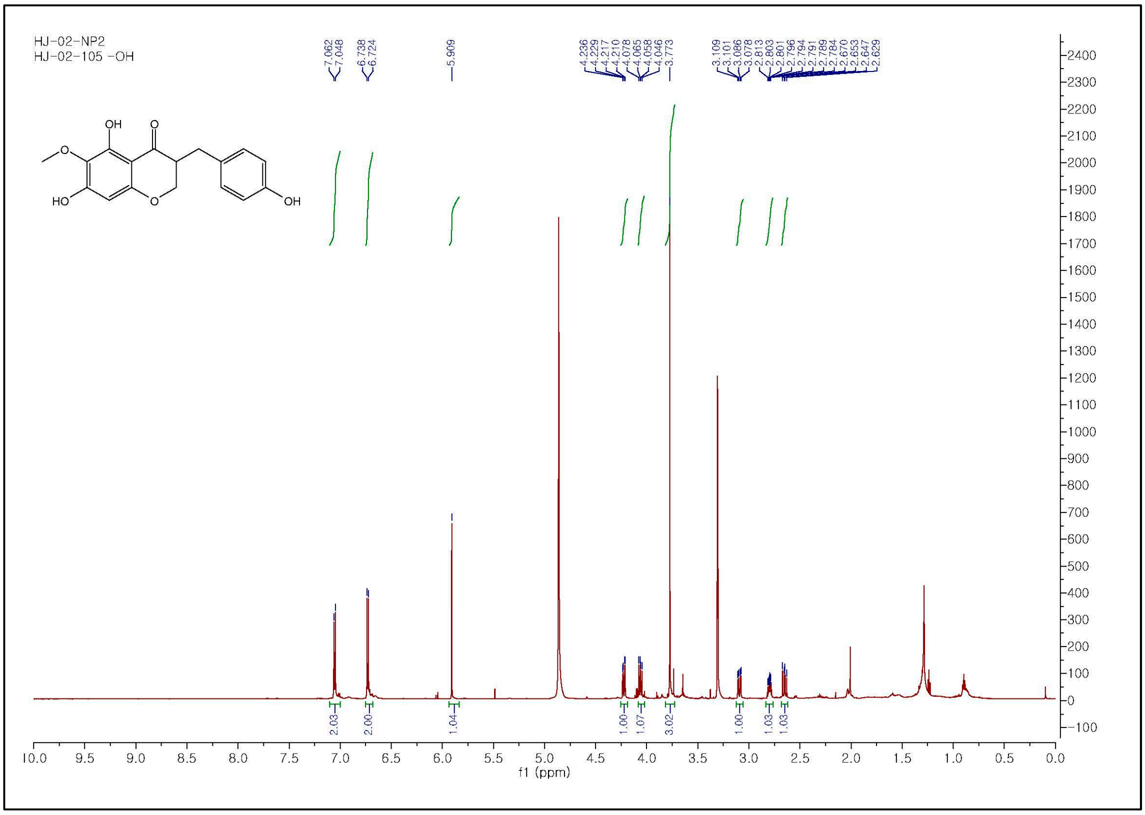

Figure S25. ^{1}H-NMR (600 MHz, CD_3OD) of compound **4**.

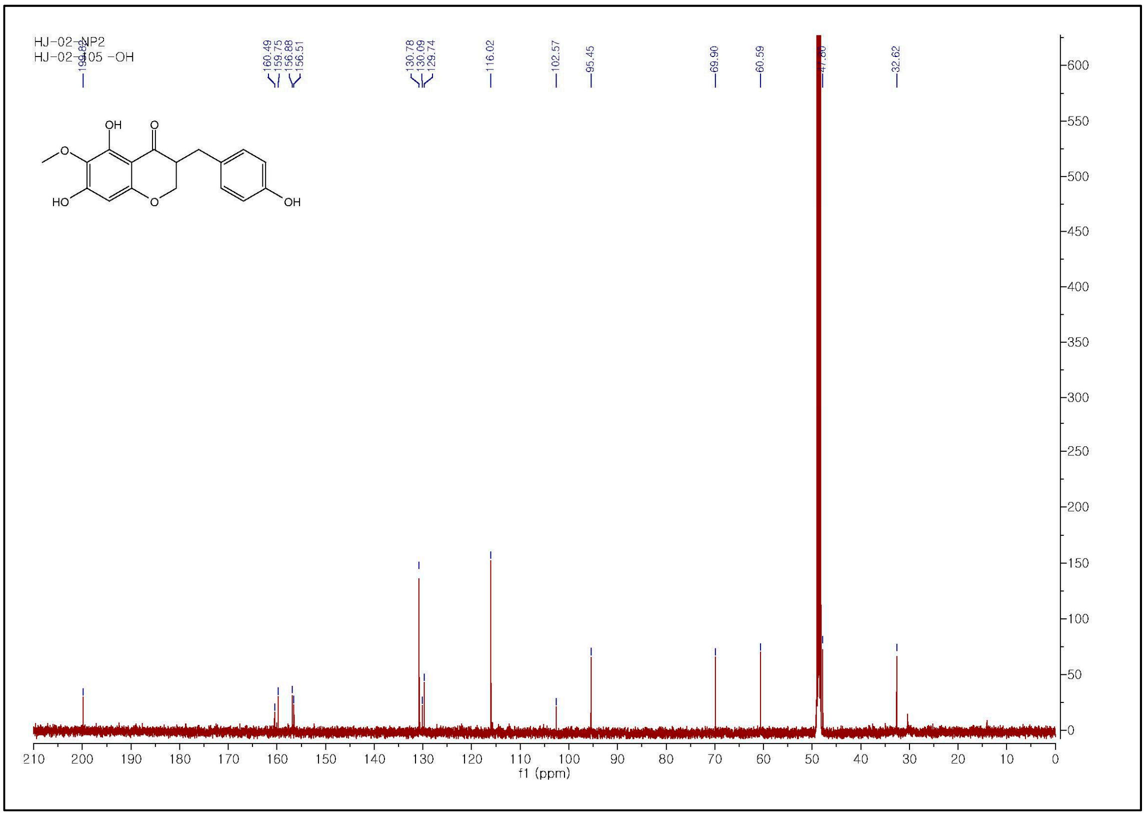

Figure S26. ^{13}C-NMR (150 MHz, CD_3OD) of compound **4**.

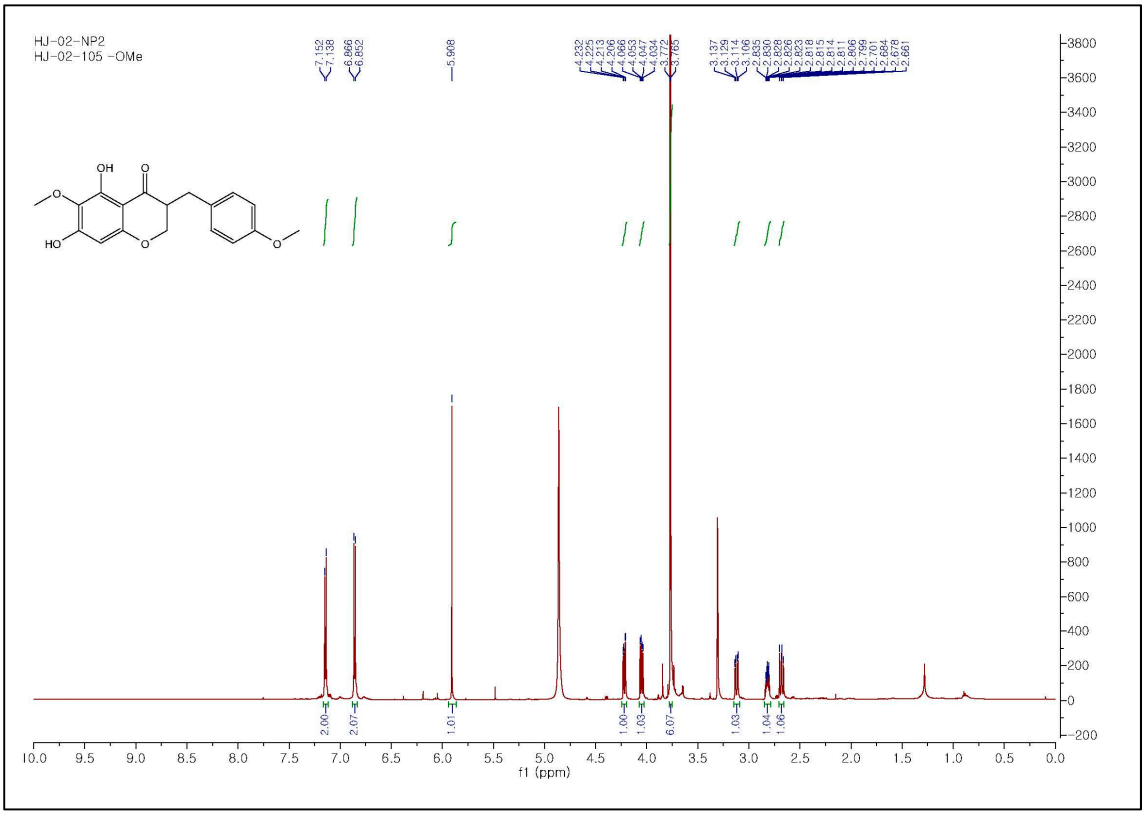

Figure S27. ^{1}H-NMR (600 MHz, CD_3OD) of compound **5**.

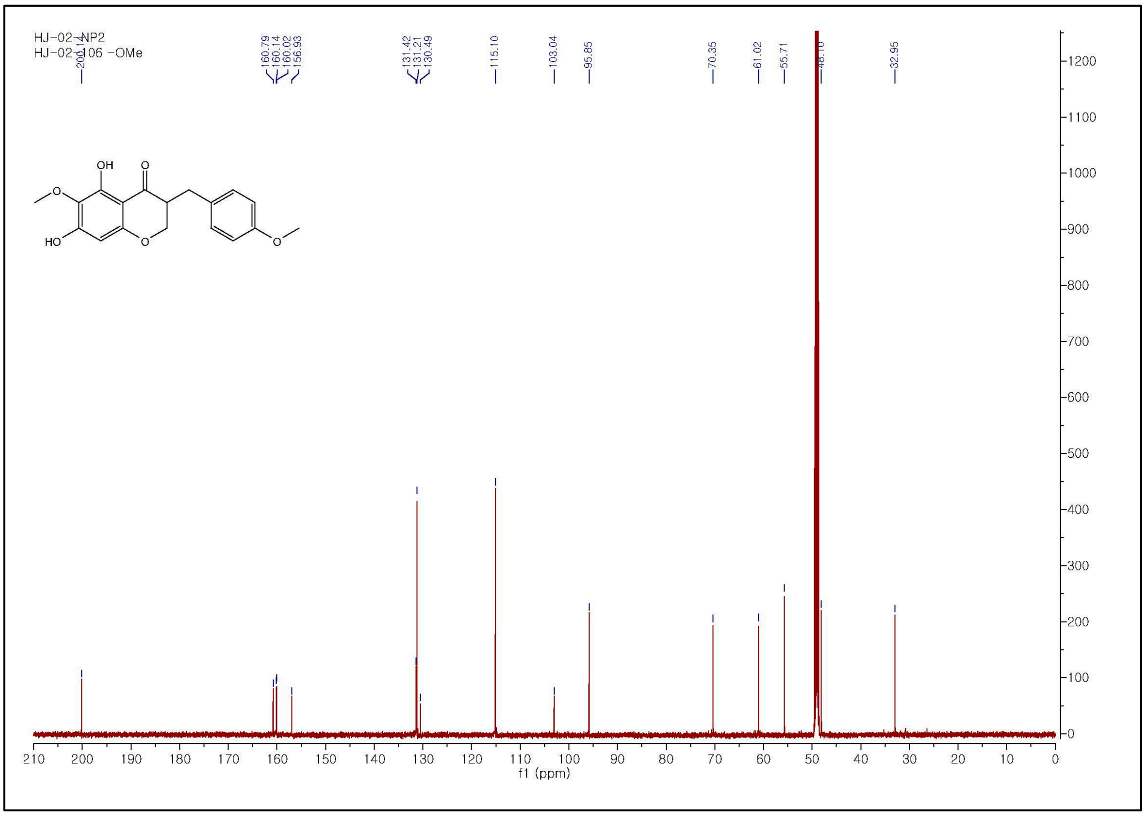

Figure S28. ^{13}C-NMR (150 MHz, CD_3OD) of compound **5**.

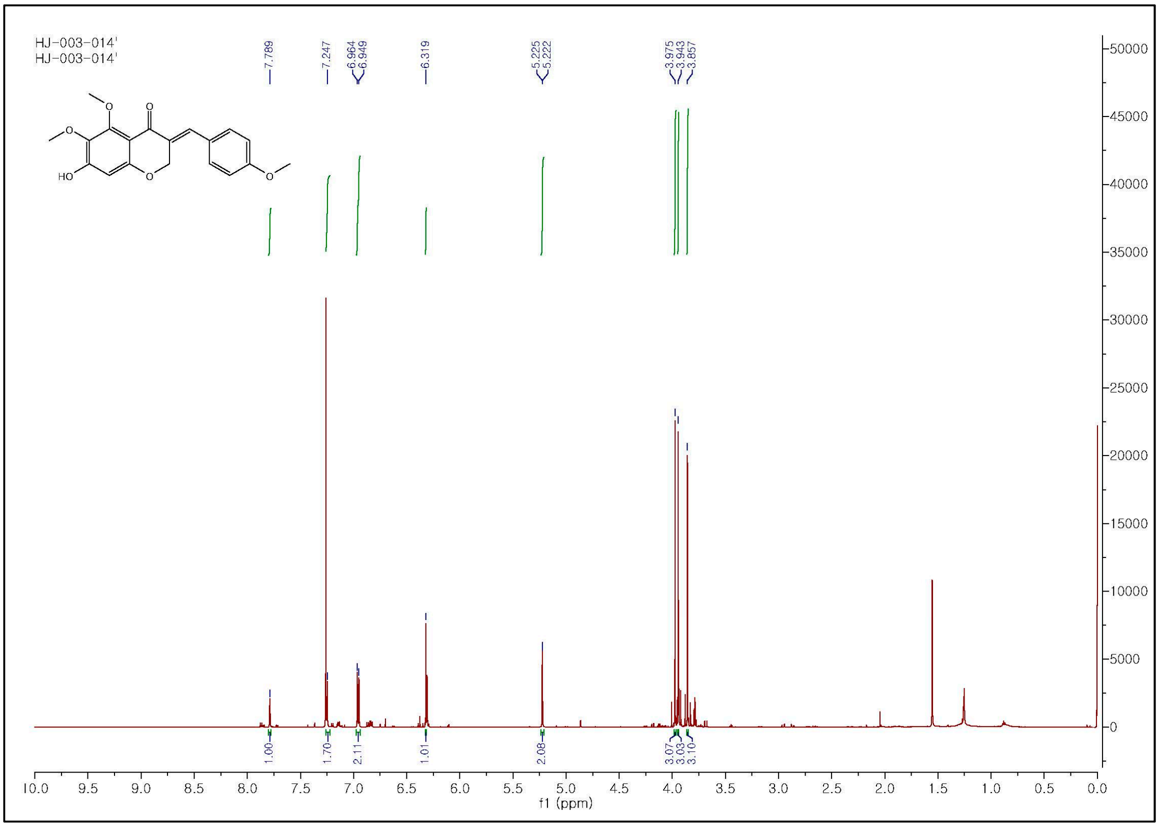

Figure S29. ^{1}H-NMR (600 MHz, $CDCl_3$) of compound **19**.

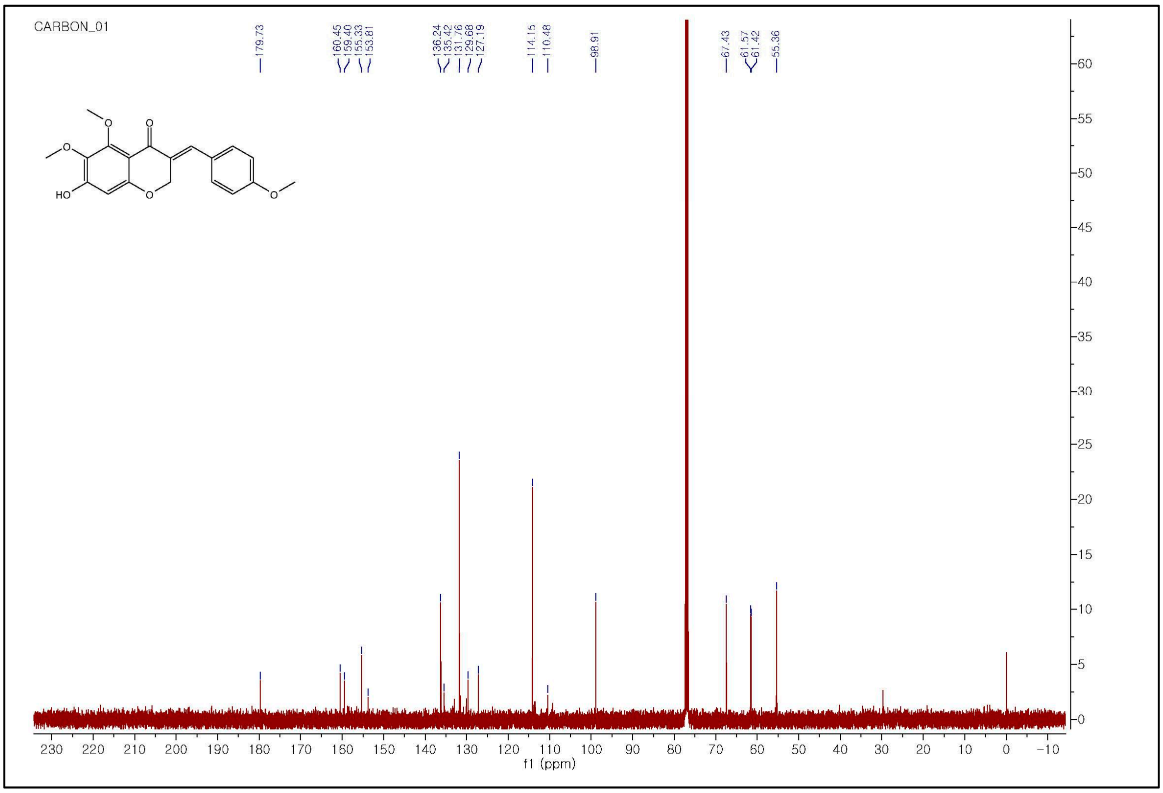

Figure S30. ^{13}C-NMR (100 MHz, $CDCl_3$) of compound **19**.

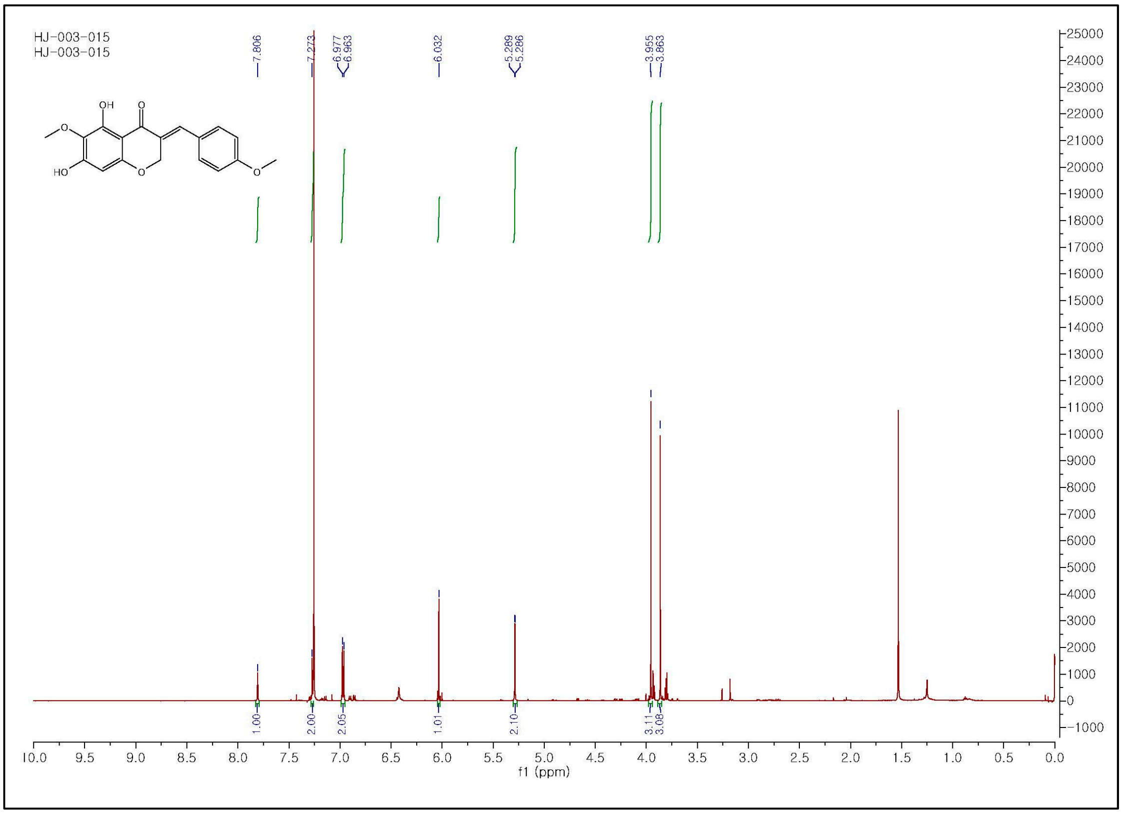

Figure S31. ^{1}H-NMR (600 MHz, $CDCl_3$) of compound **8**.

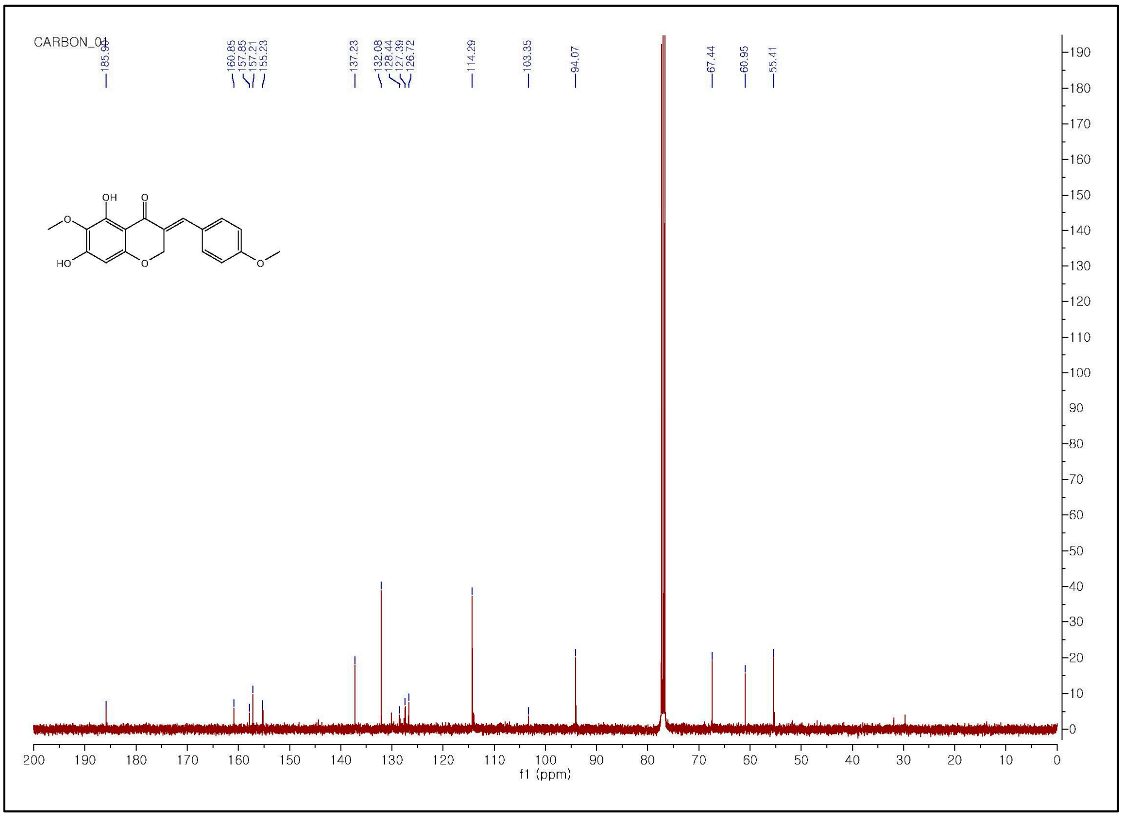

Figure S32. ^{13}C-NMR (100 MHz, $CDCl_3$) of compound **8**.
